# Supplementary material for: Transient APC/C inactivation by mTOR boosts glycolysis during cell cycle entry
Source: Nature. 2025 Jul 30;646(8083):198–207. doi: 10.1038/s41586-025-09328-w (PMC12488482; doi:10.1038/s41586-025-09328-w)

nature portfolio

https://doi.org/10.1038/s41586-025-09328-w

Supplementary information

Transient APC/C inactivation by mTOR boosts glycolysis during cell cycle entry

In the format provided by the authors and unedited

Fig. 2d

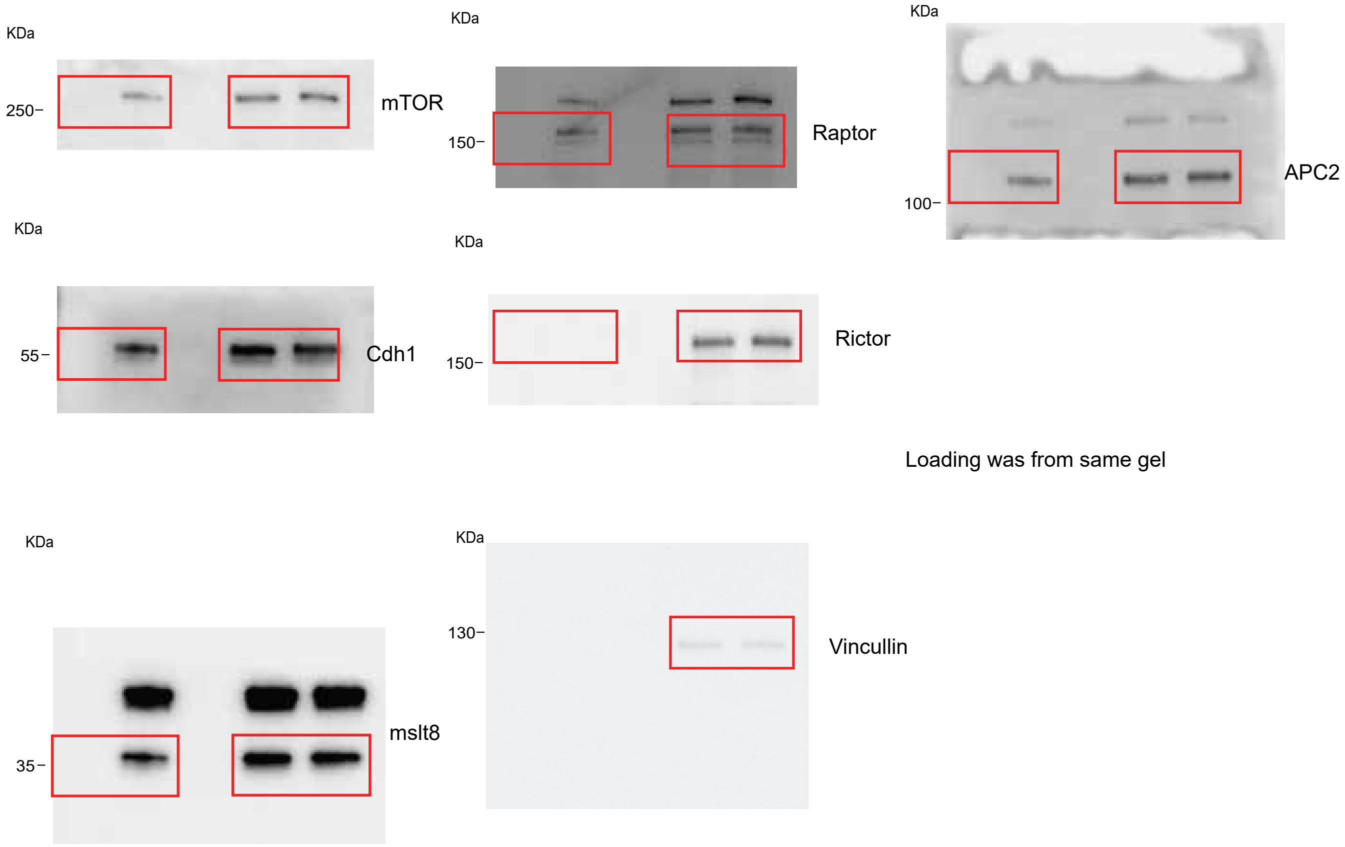

Fig. 2e

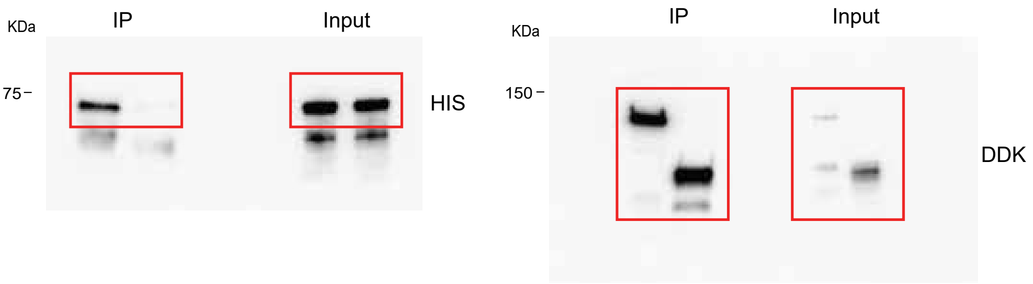

Fig. 2f

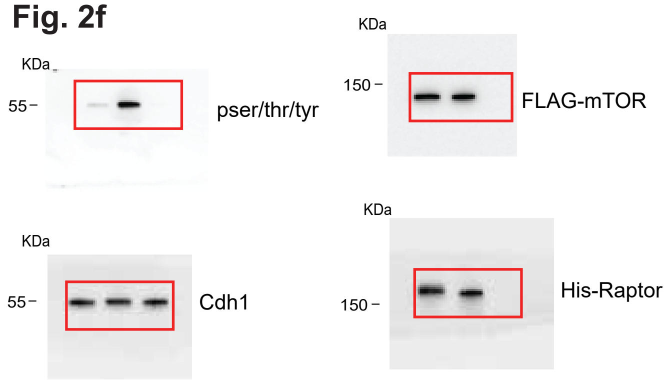

Fig. 2g

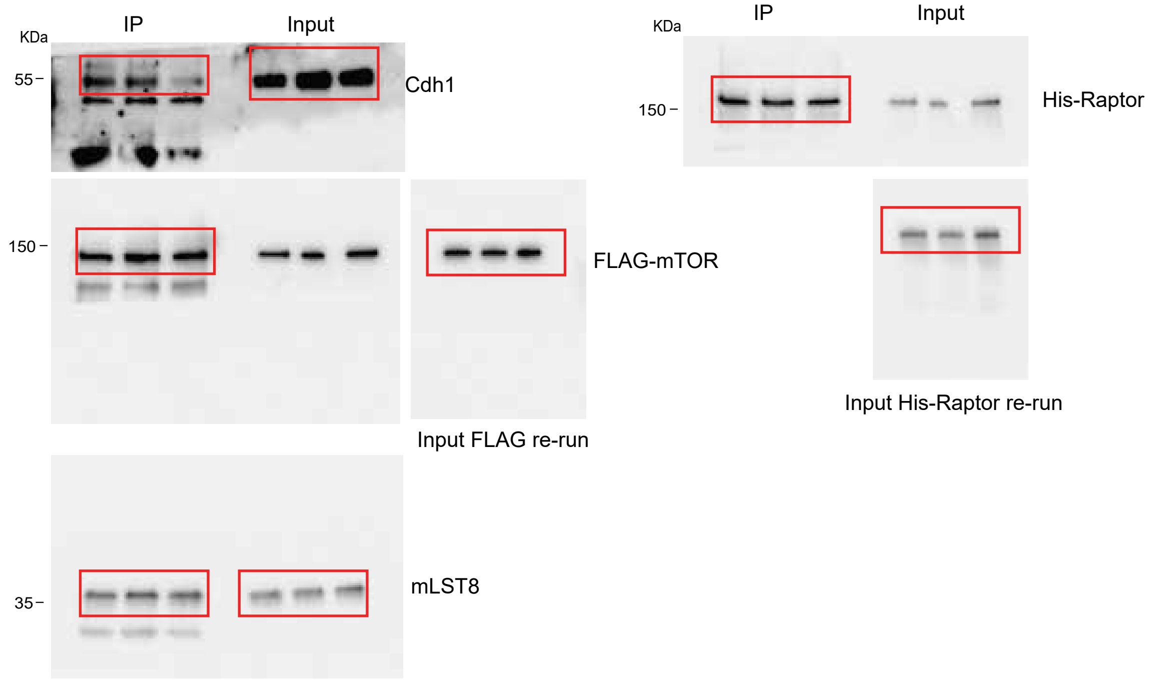

Fig. 2h

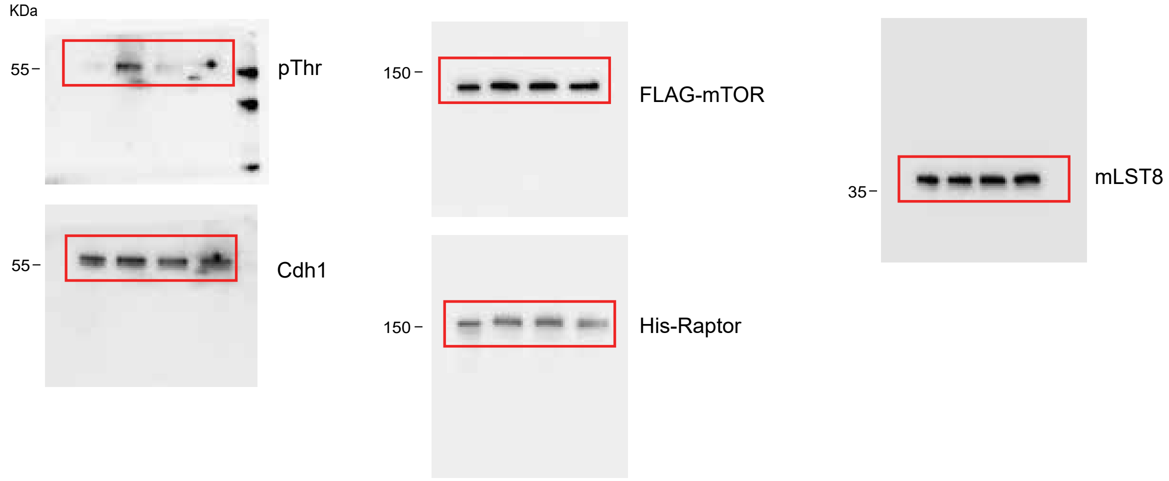

Fig. 2j

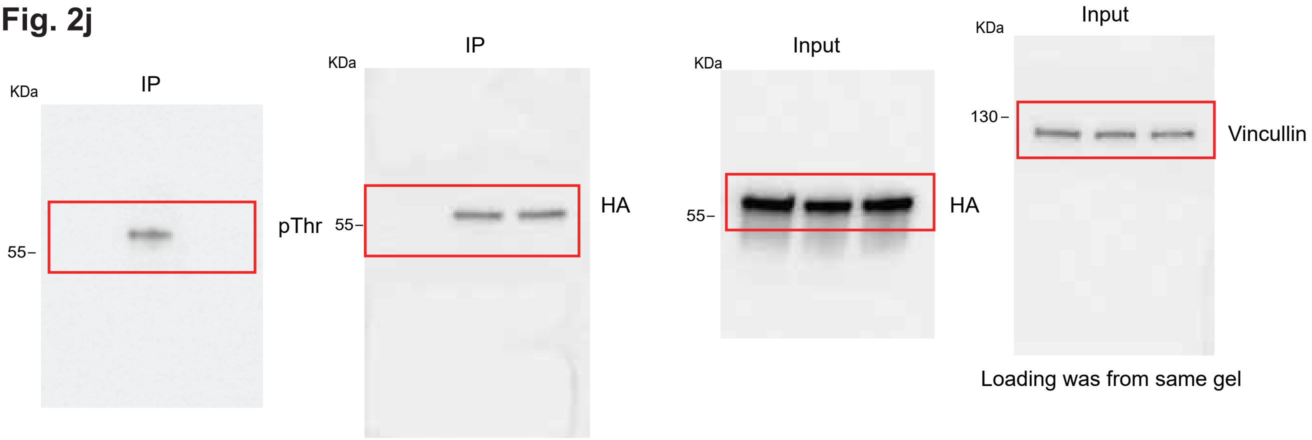

Loading was from same gel

Fig. 2k

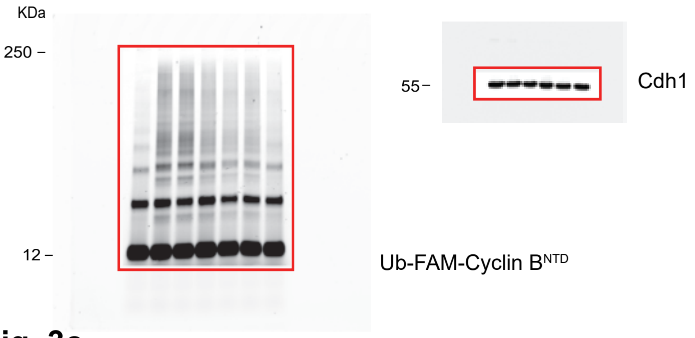
KDa
250 –
12 –
55 –
Cdh1
Ub-FAM-Cyclin B^{NTD}

Fig. 3c

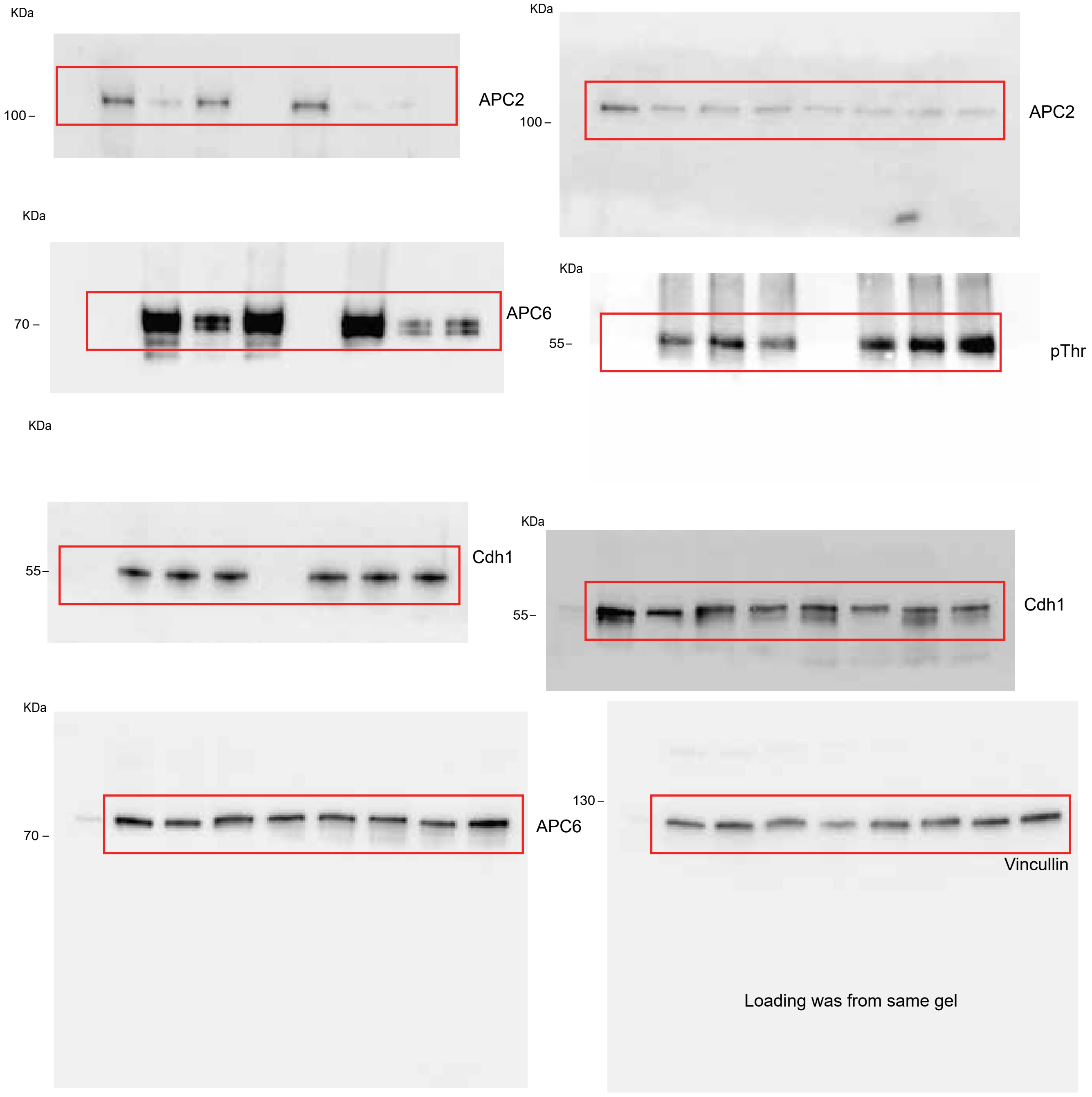
KDa
100 –
APC2
KDa
100 –
APC2
KDa
70 –
APC6
KDa
55 –
pThr
KDa
55 –
Cdh1
KDa
55 –
Cdh1
KDa
70 –
APC6
130 –
Vincullin
Loading was from same gel

Fig. 3d

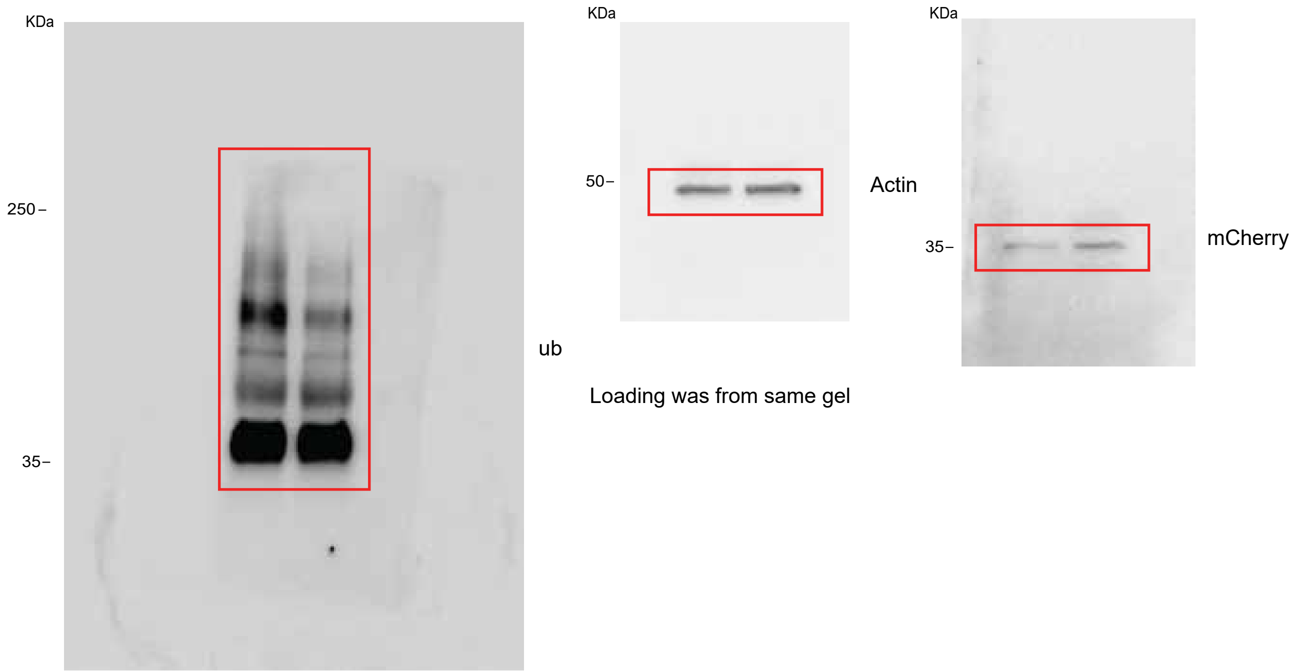

Loading was from same gel

Fig. 4a

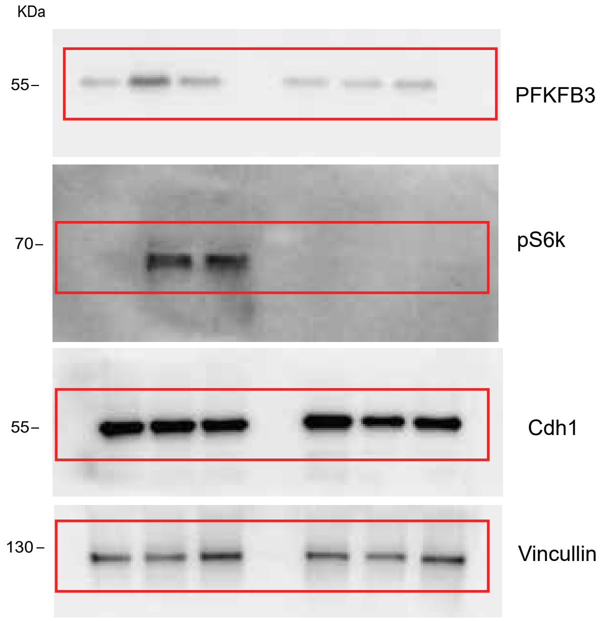

Loading was from same gel

Fig. 4b

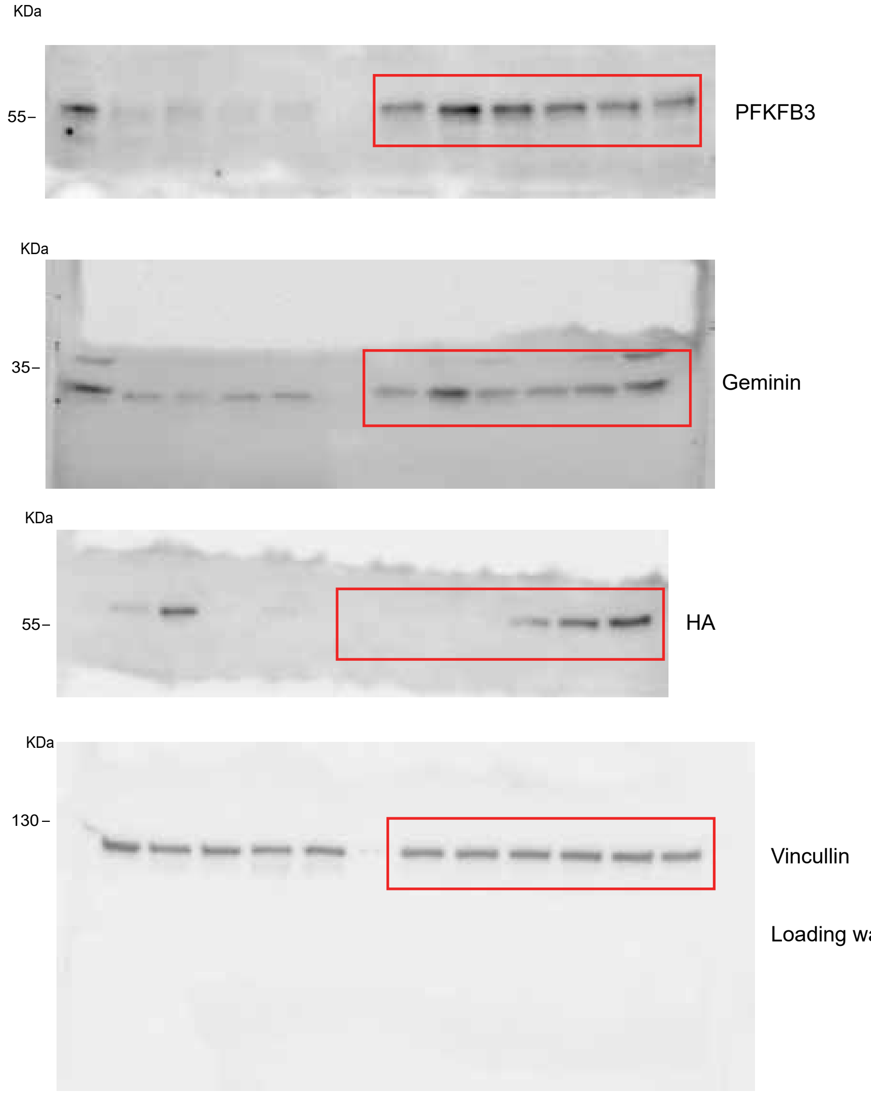

Loading was from same gel

Fig. 4c

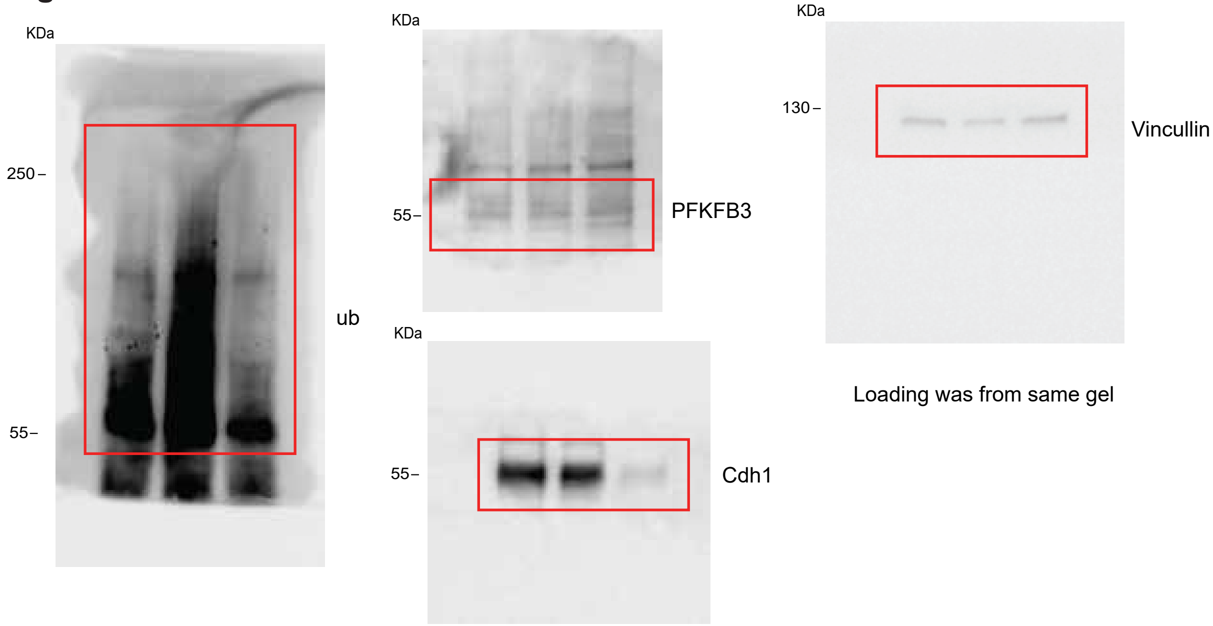

Loading was from same gel

Fig. 4d

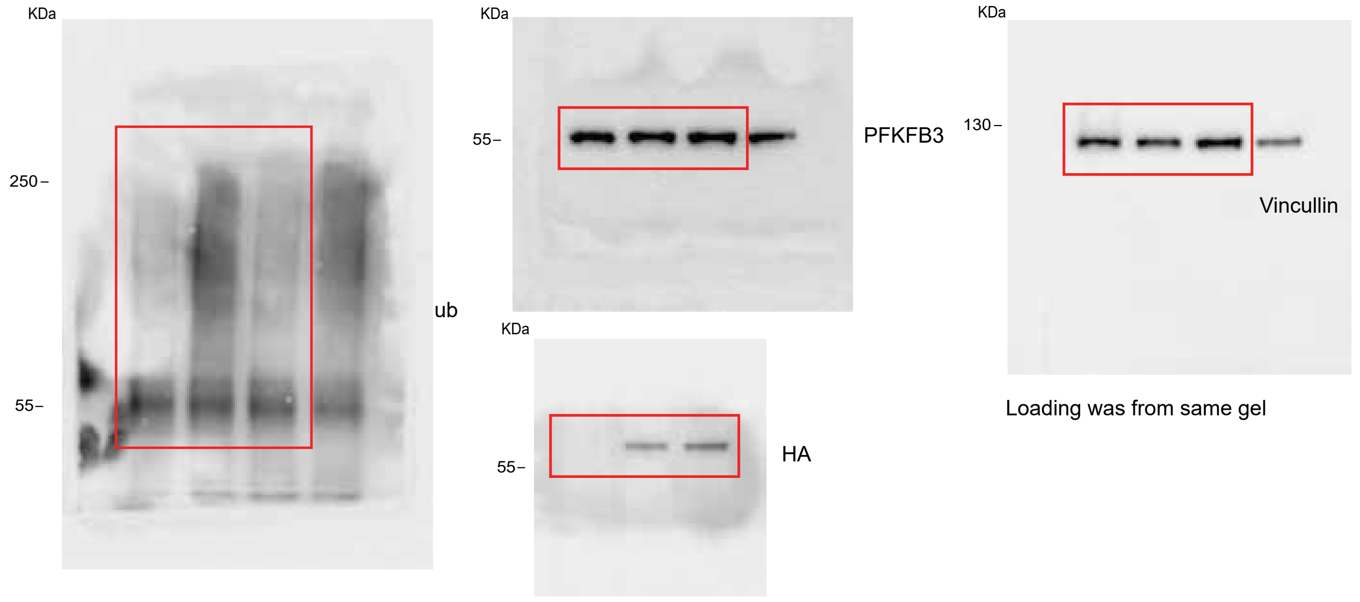

HA: Same input (only first 3 samples)
re-ran and robed for HA in a separate blot

Fig. 4e

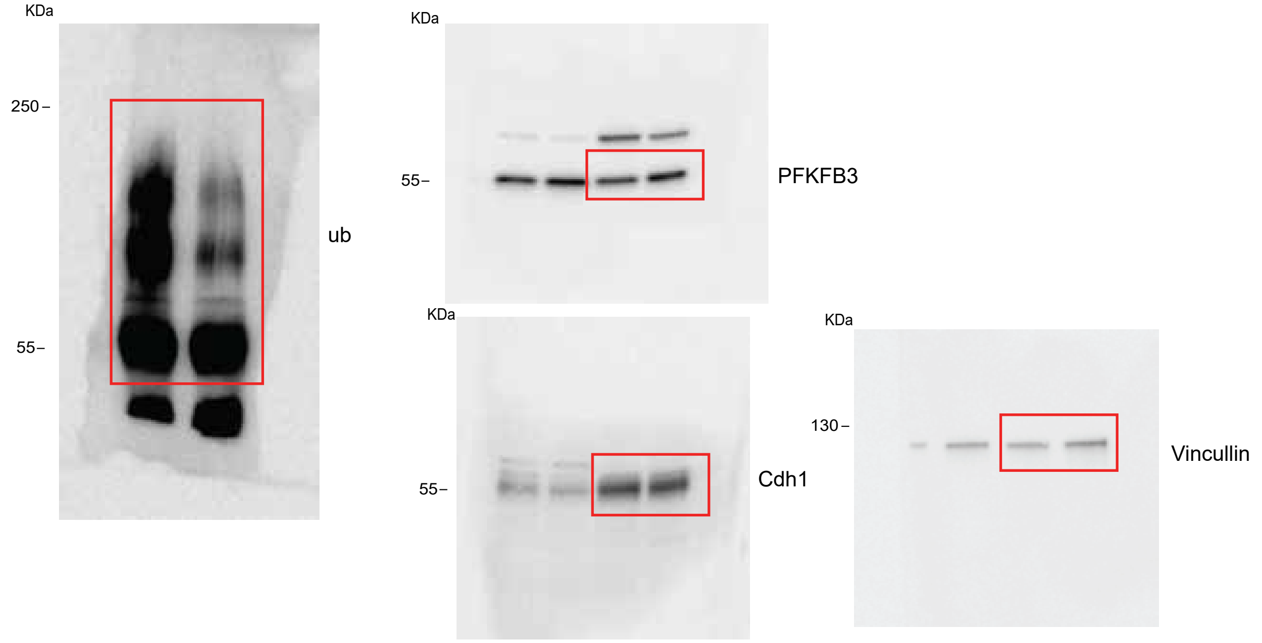

Loading was from same gel

Extended Data Fig. 1a

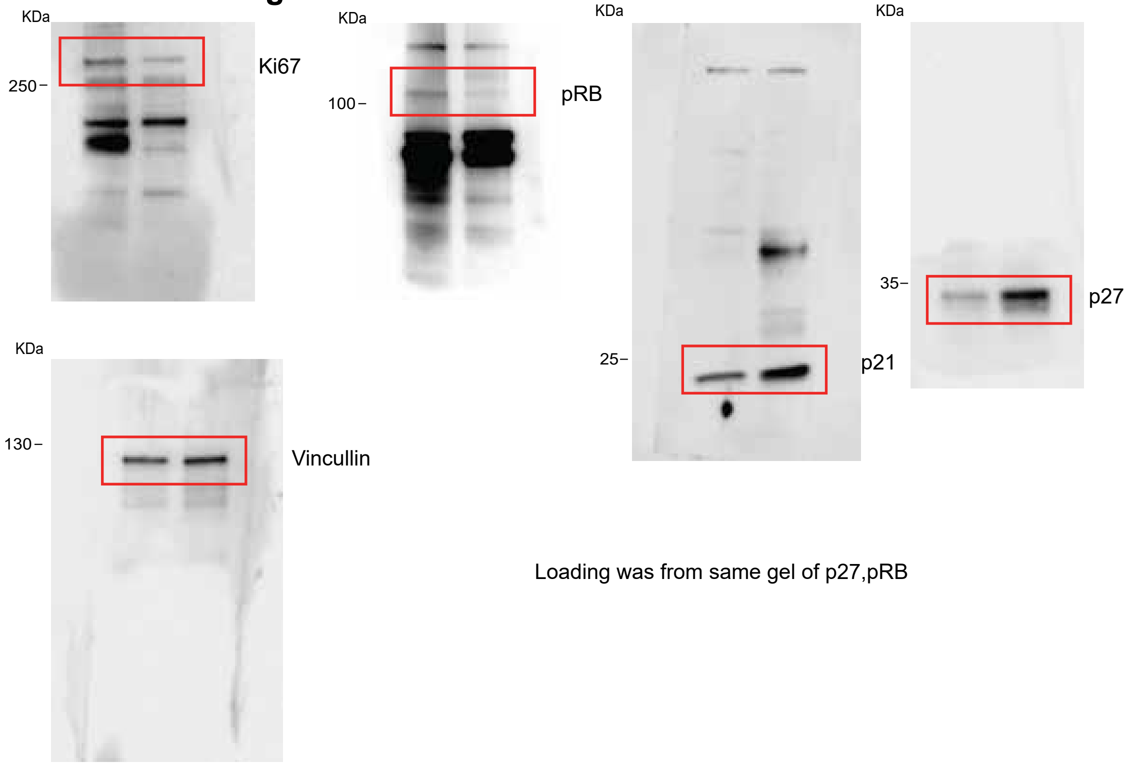

Loading was from same gel of p27,pRB

Extended Data Fig. 1g

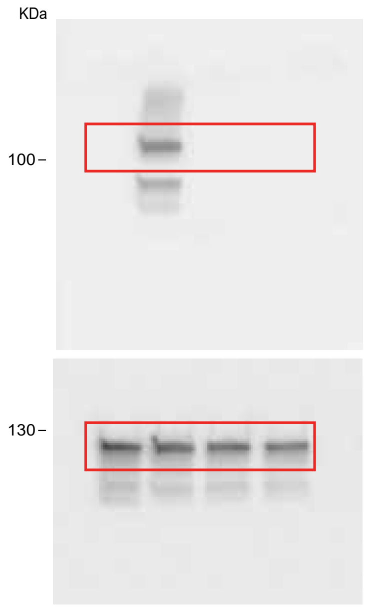

Loading from same gel

Extended Data Fig. 1h

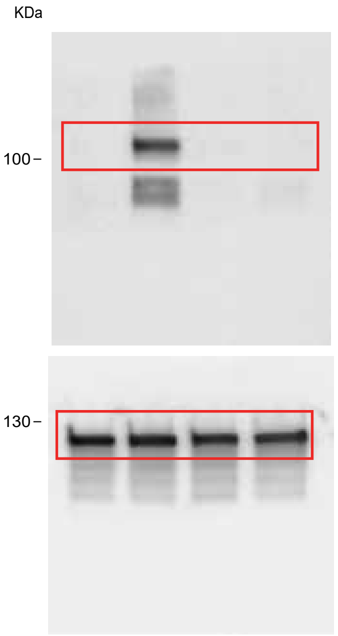

Loading from same gel

Extended Data Fig. 1i

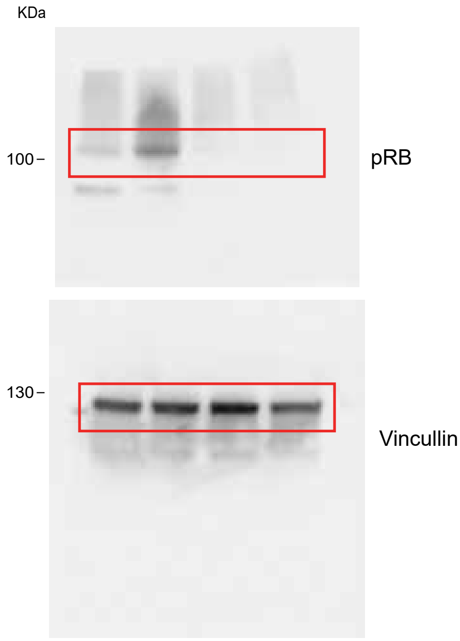

Loading from same gel

Extended Data Fig. 2h

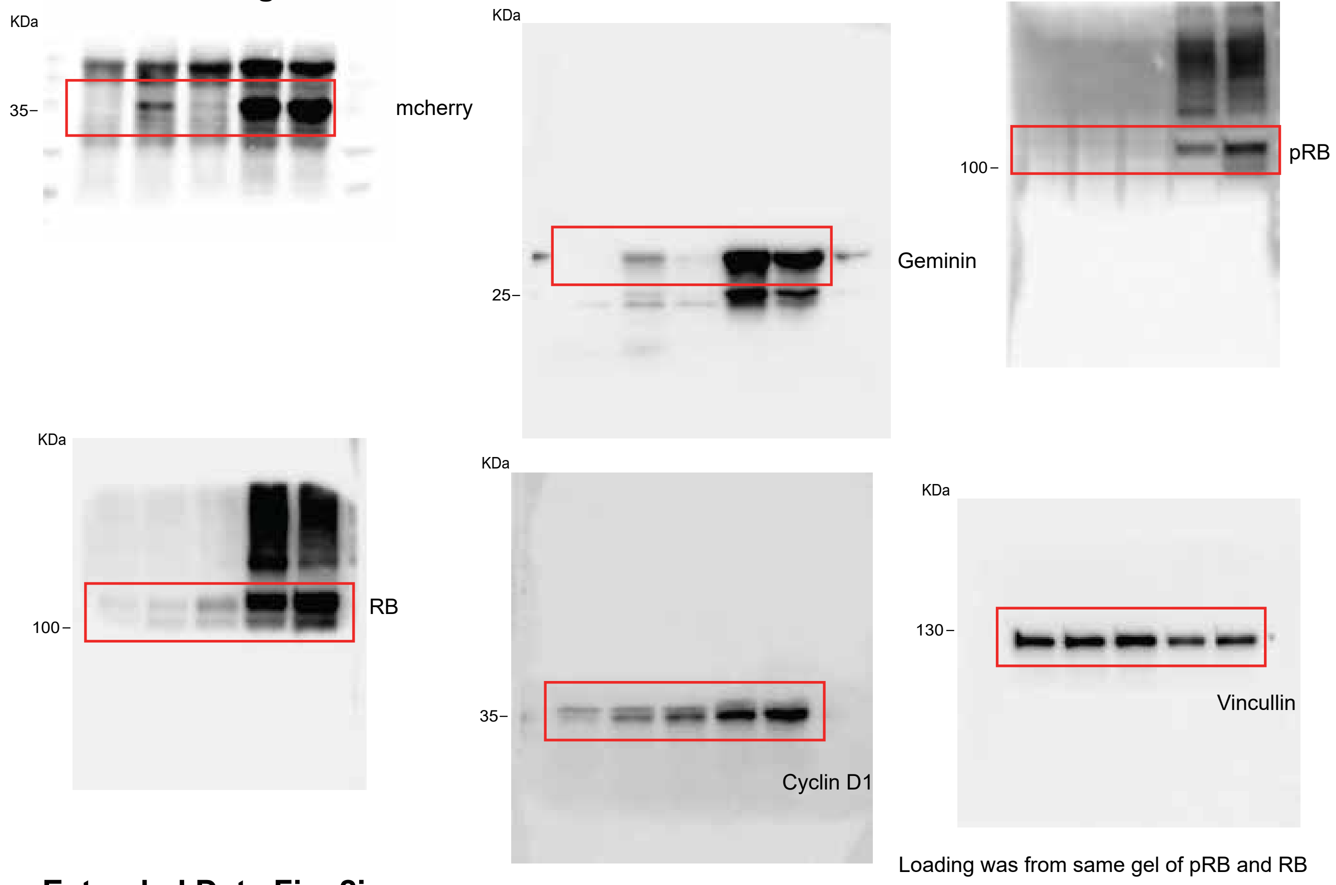

Extended Data Fig. 2j

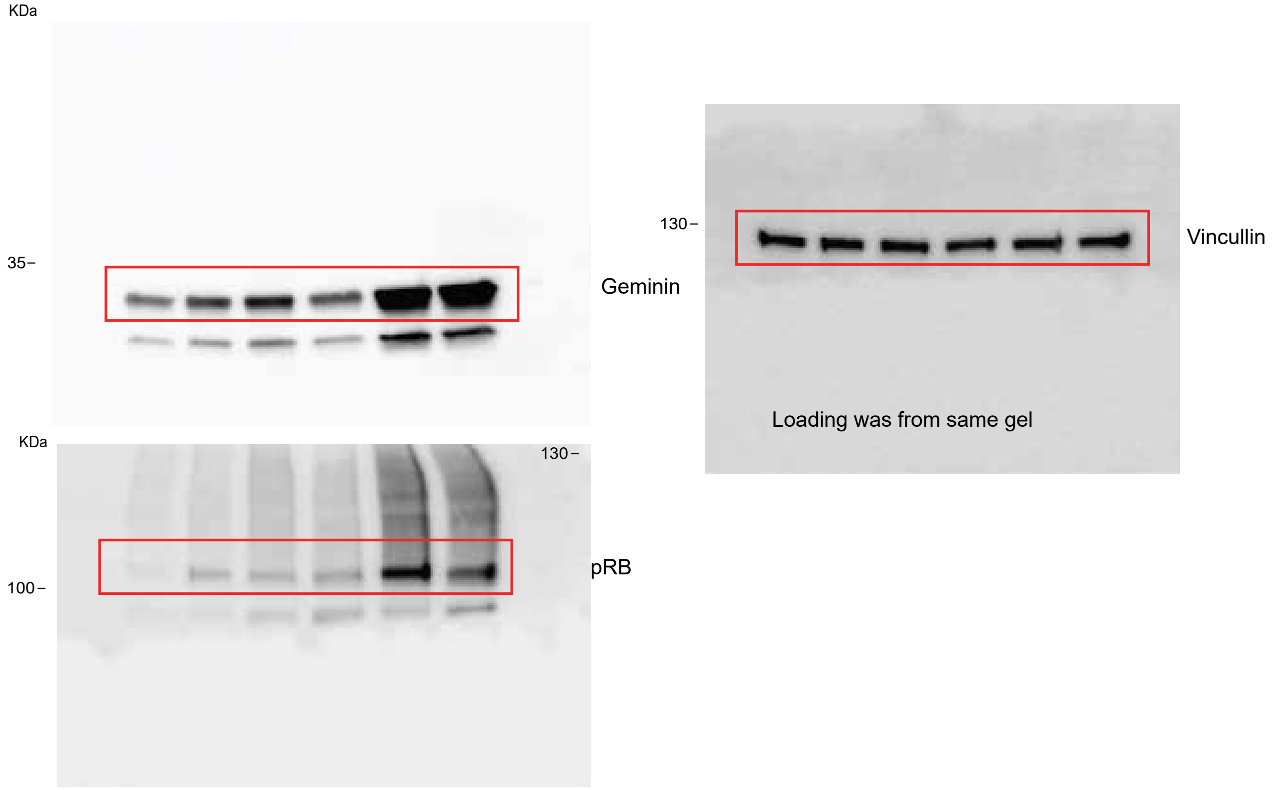

Extended Data Fig. 2k

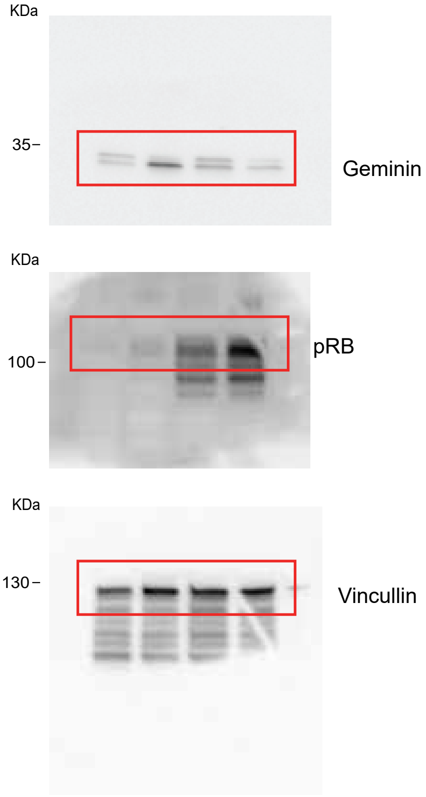

Loading was from same gel

Extended Data Fig. 2l

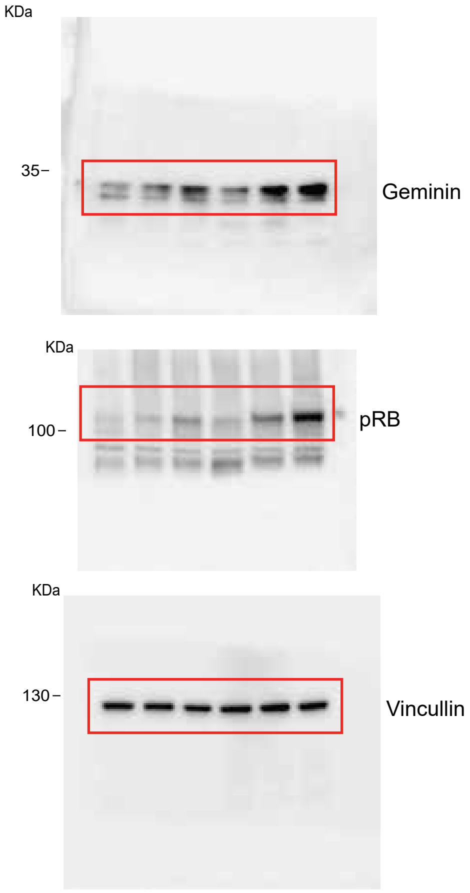

Loading was from same gel

Extended Data Fig. 3b

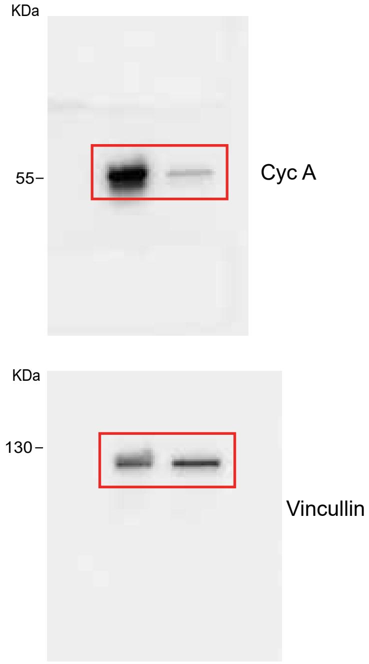

Loading was from same gel

Extended Data Fig. 3c

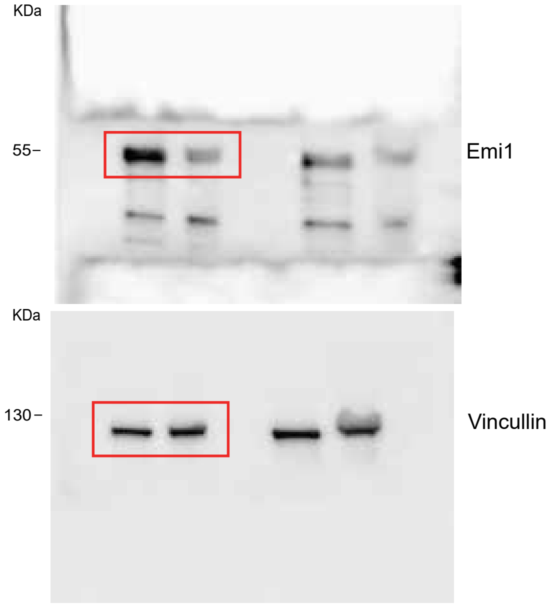

Loading was from same gel

Extended Data Fig. 3d

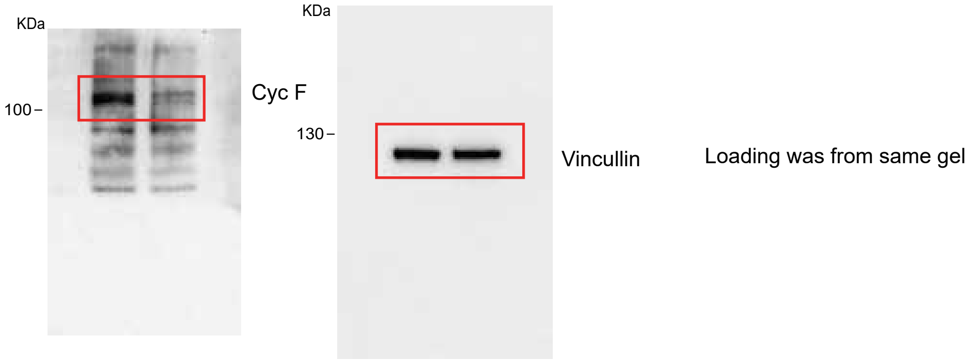

Extended Data Fig. 3e

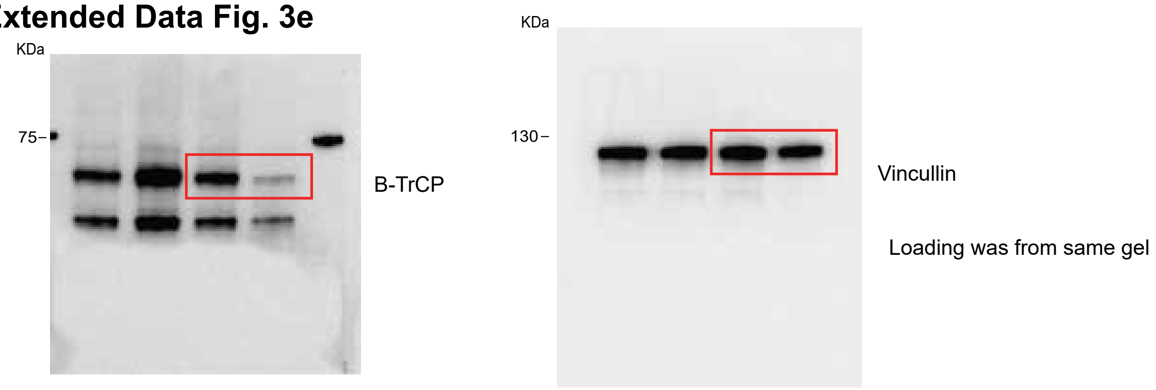

Extended Data Fig. 3k

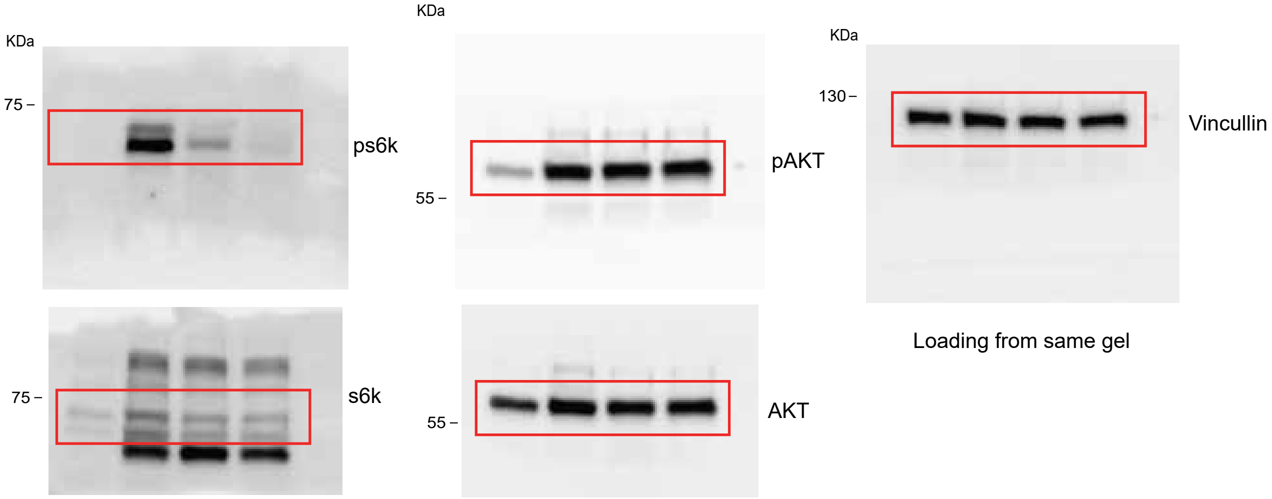

Extended Data Fig. 3m

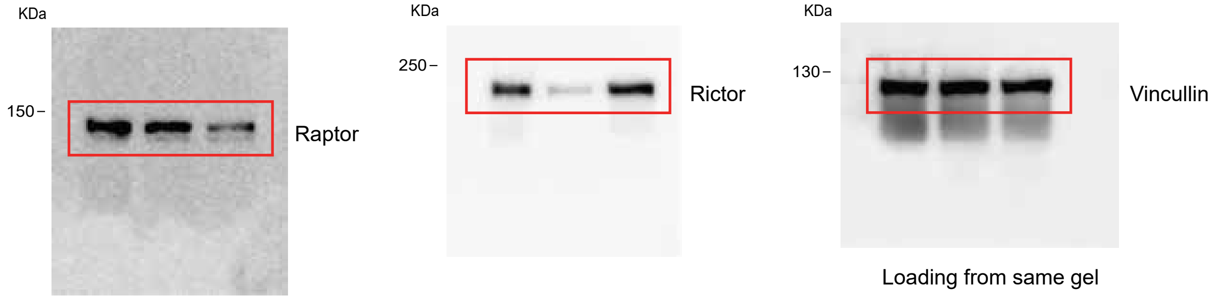

Extended Data Fig. 4a

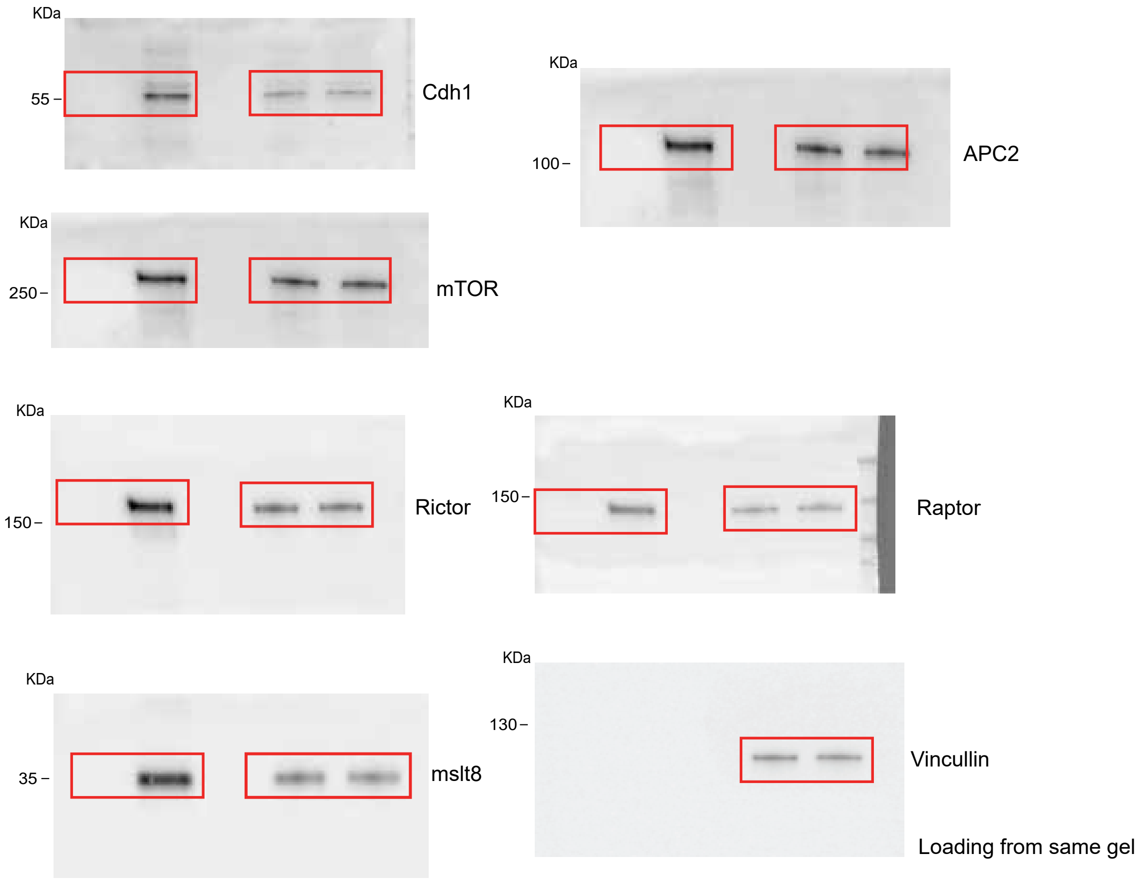

Extended Data Fig. 4b

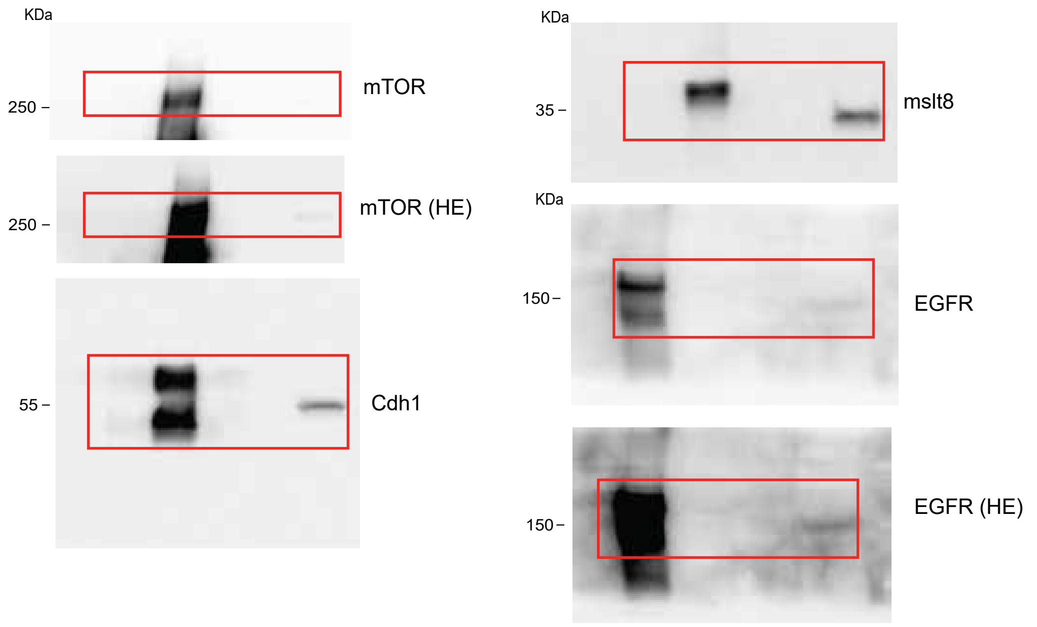

Extended Data Fig. 4c

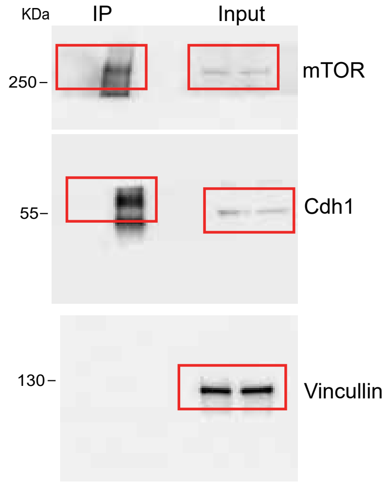

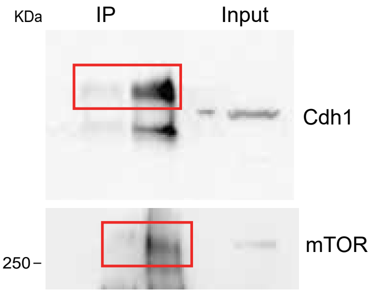

Loading from same gel

Extended Data Fig. 4d

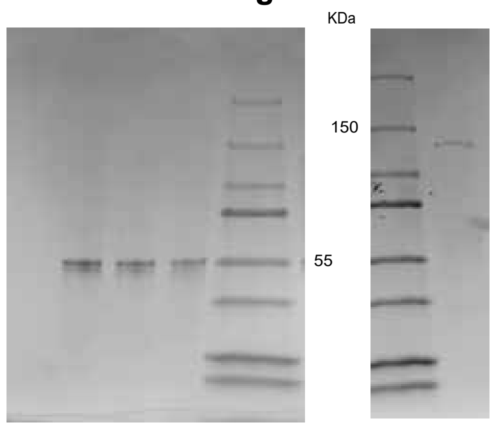

Extended Data Fig. 4e

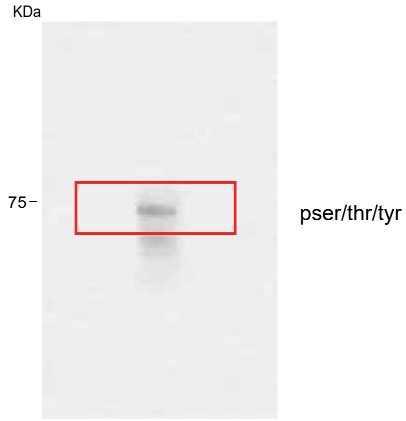

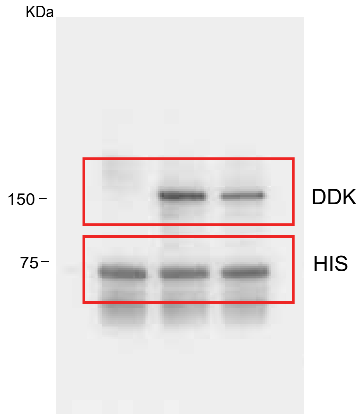

Extended Data Fig. 4f

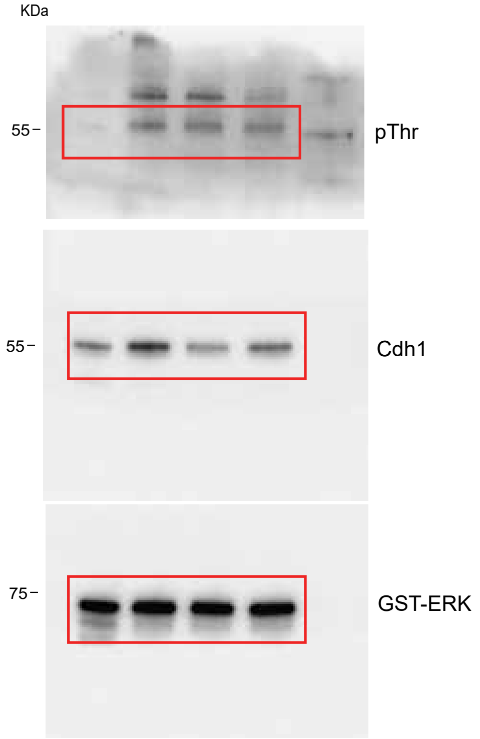

Extended Data Fig. 4g

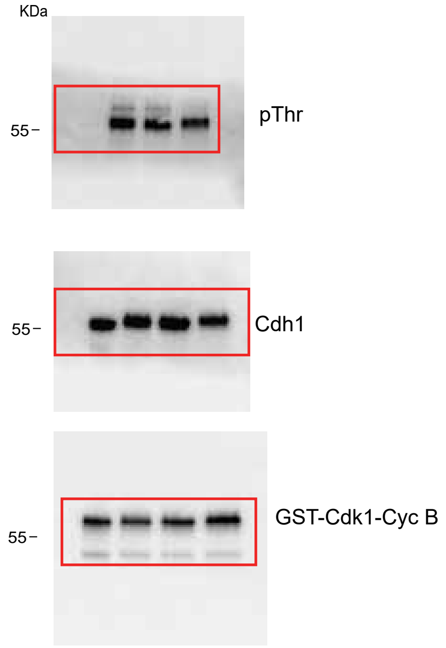

Extended Data Fig. 4h

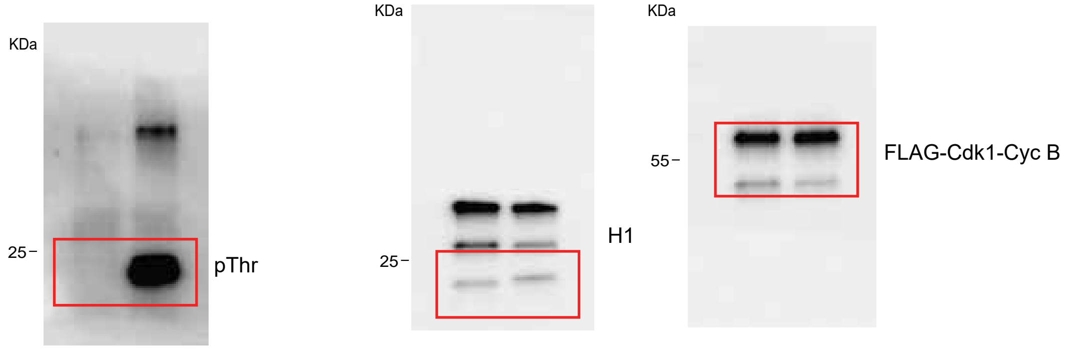

Extended Data Fig. 4i

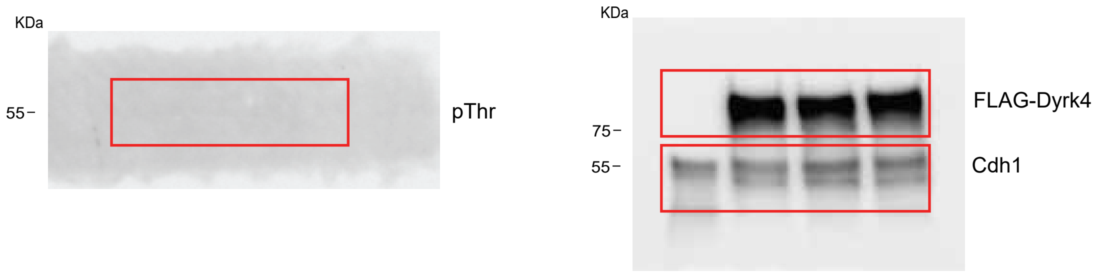

Extended Data Fig. 4j

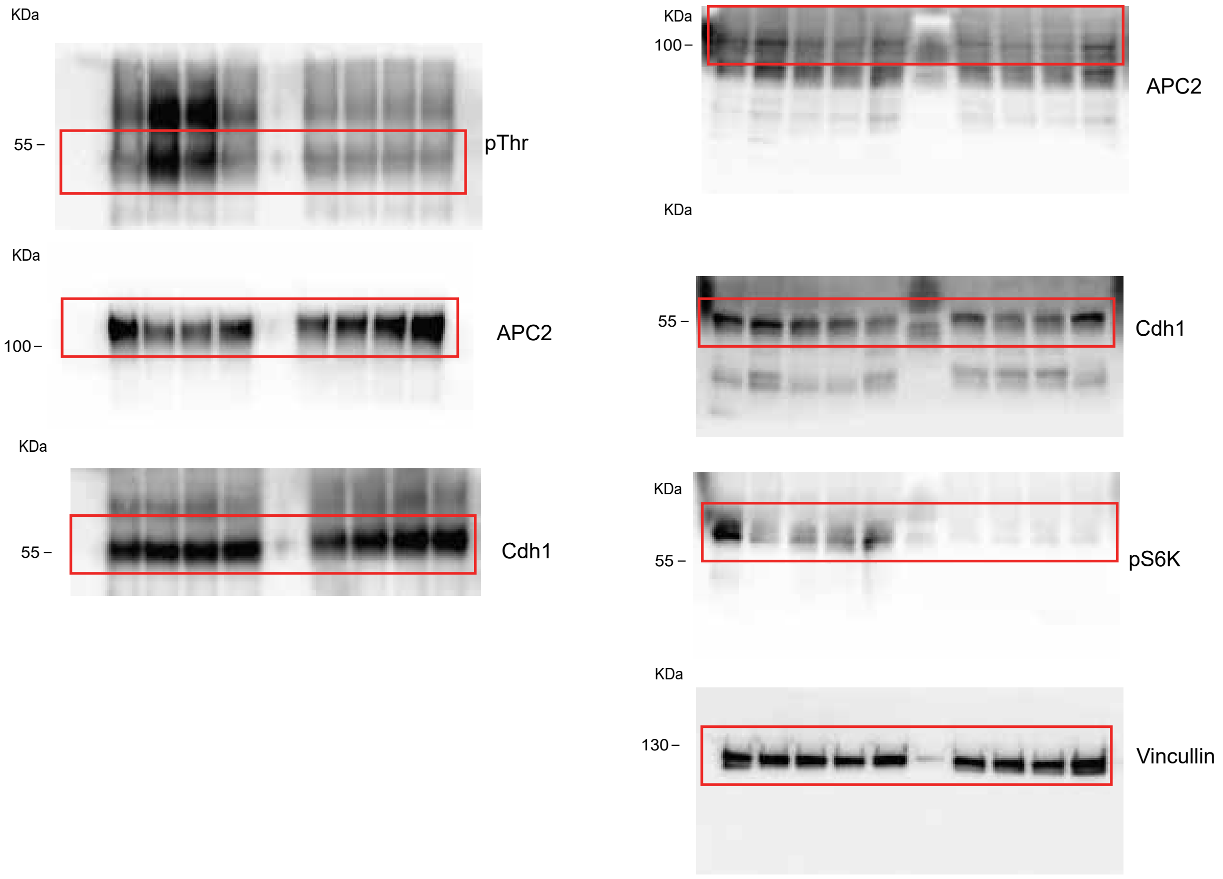

Loading from same gel

Extended Data Fig. 5b

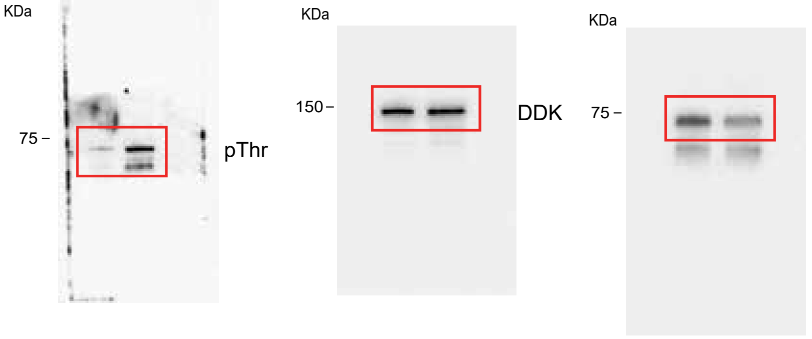

Extended Data Fig. 5c

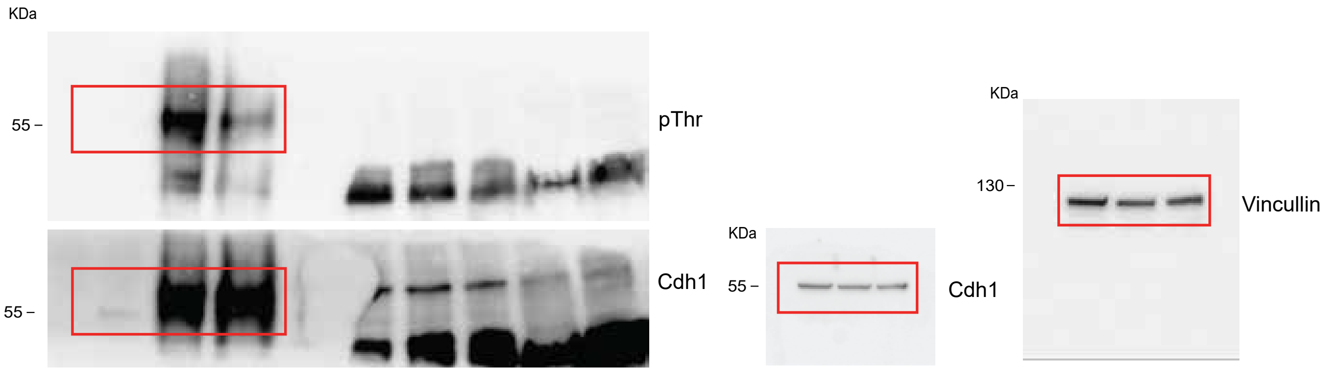

The Input samples rerun and probed for Cdh1 and Vincullin

Extended Data Fig. 5d

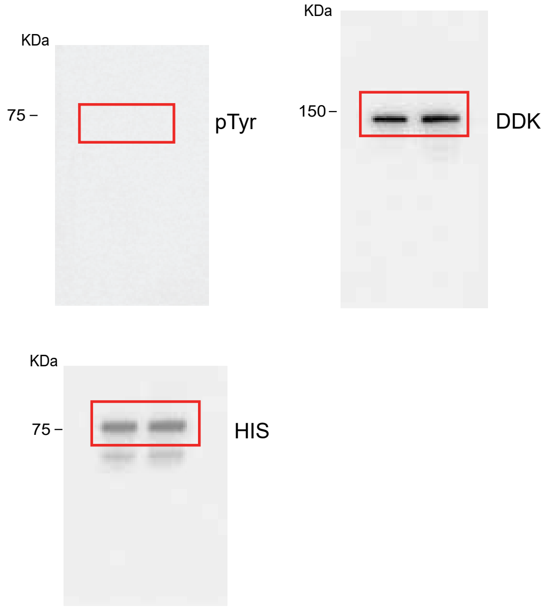

Extended Data Fig. 5e

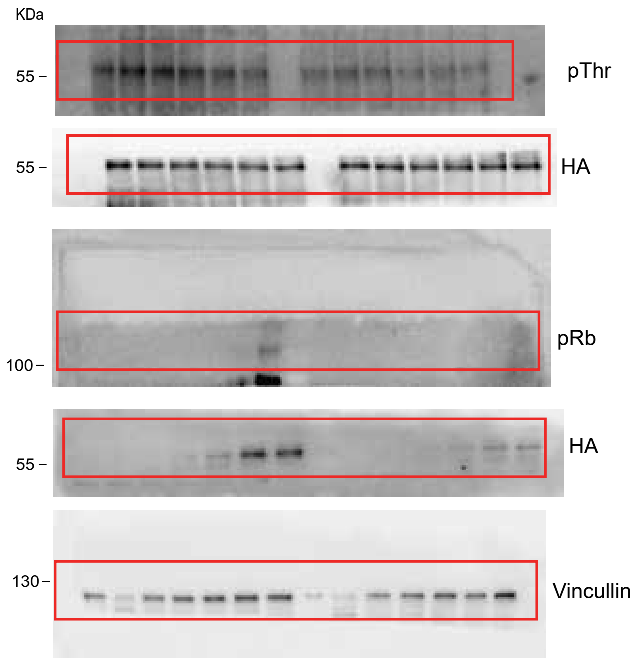

Loading from same gel

Extended Data Fig. 5h

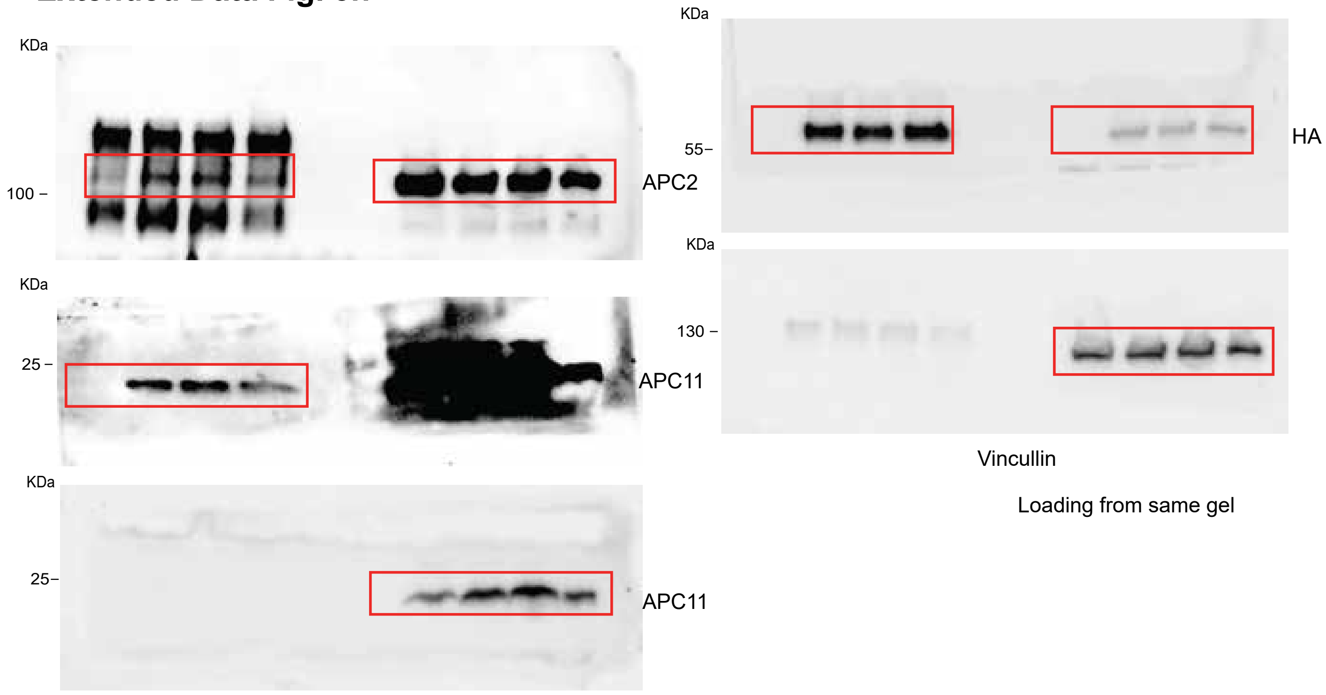

Extended Data Fig. 5j

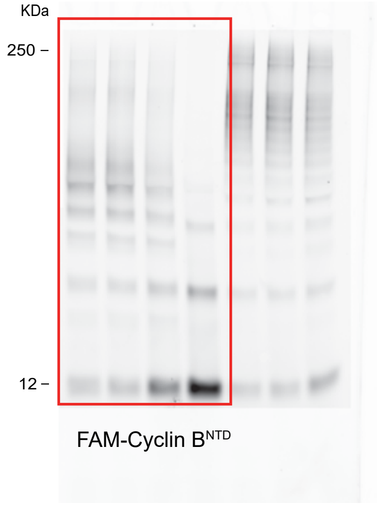

Extended Data Fig. 5k

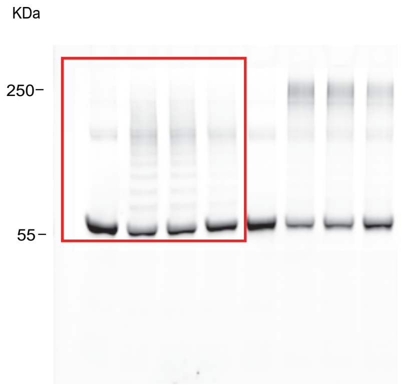

Extended Data Fig. 5o

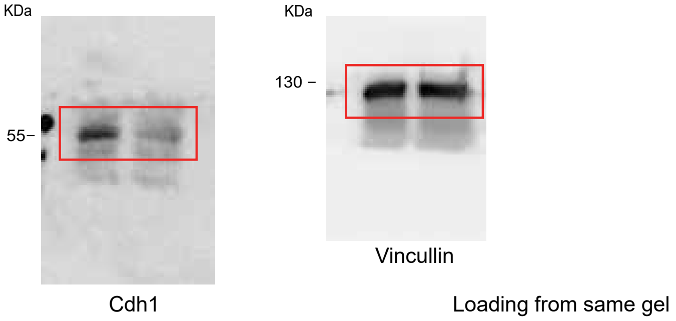

Extended Data Fig. 6a

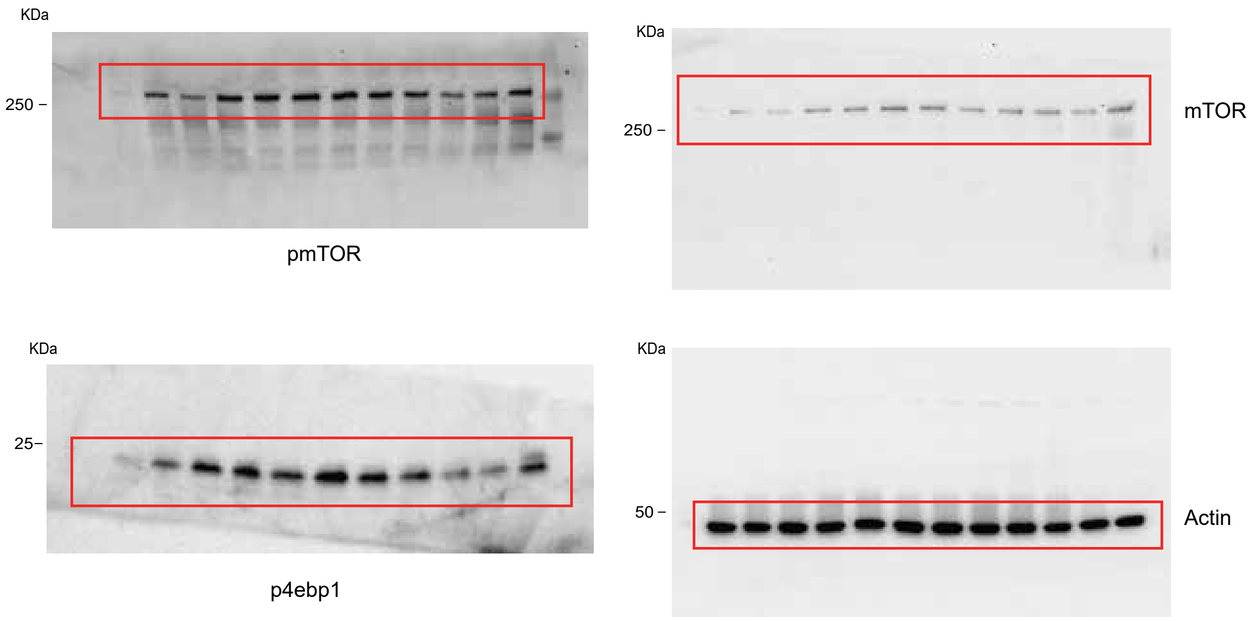

Loading from same gel

Extended Data Fig. 7a

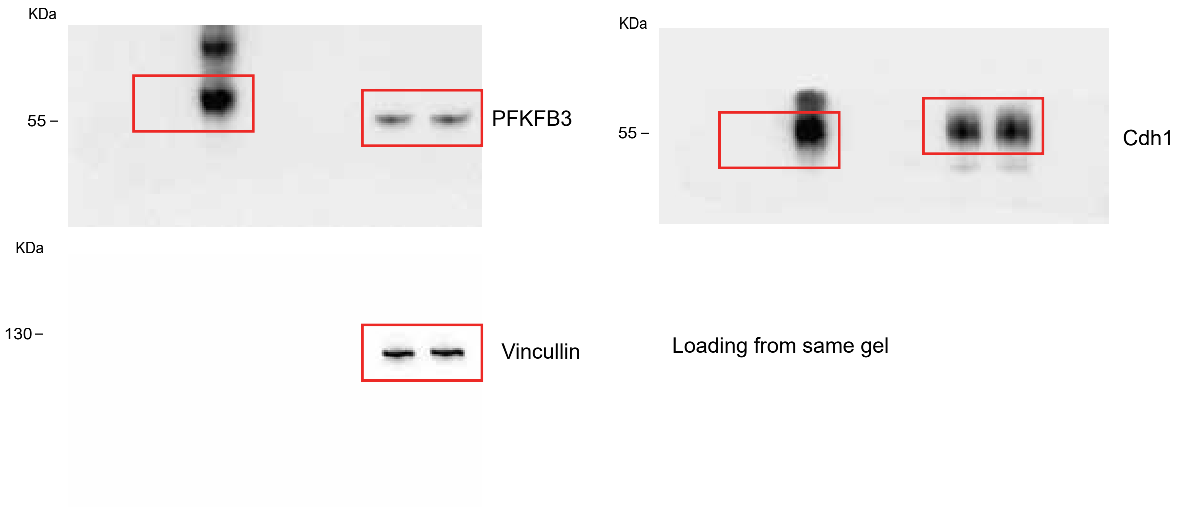

Extended Data Fig. 7b

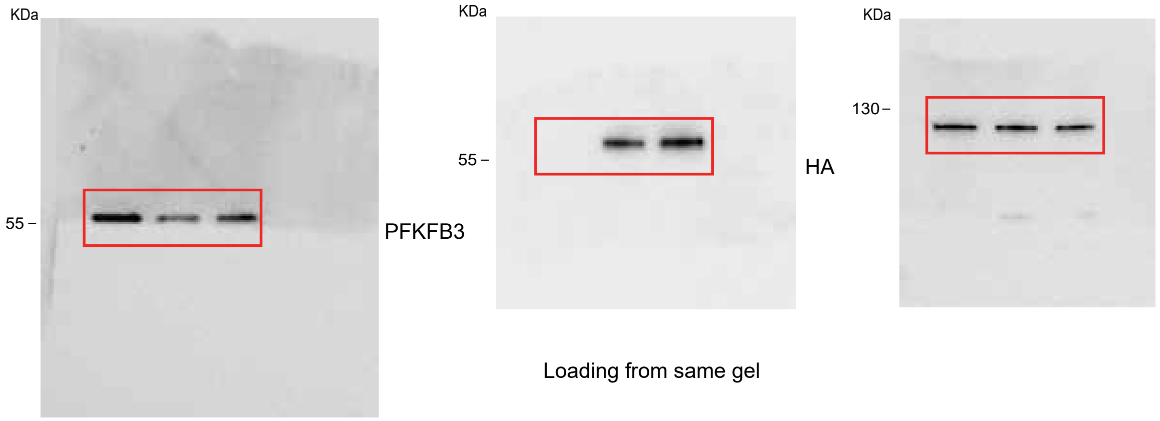

Extended Data Fig. 7c

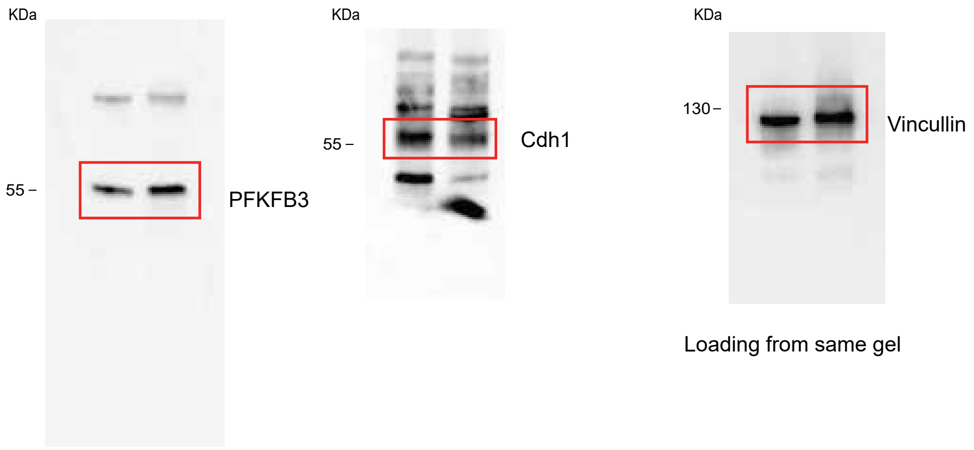

Extended Data Fig. 7d

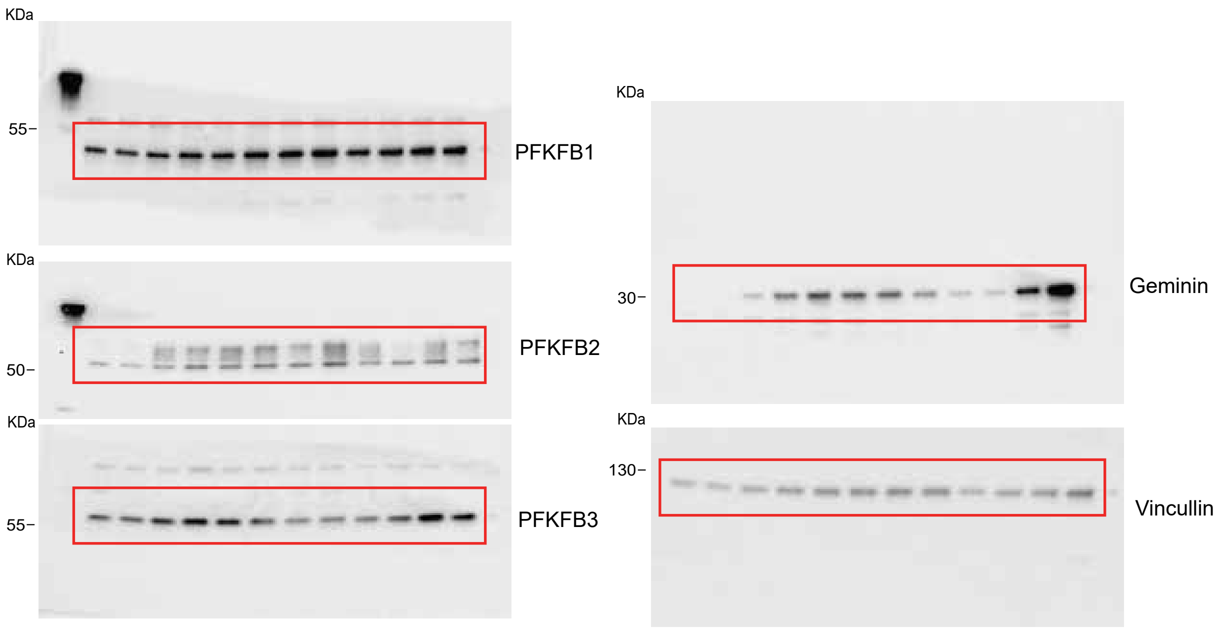

Loading from same gel, for geminin same samples were run and probed for geminin

Extended Data Fig. 7e

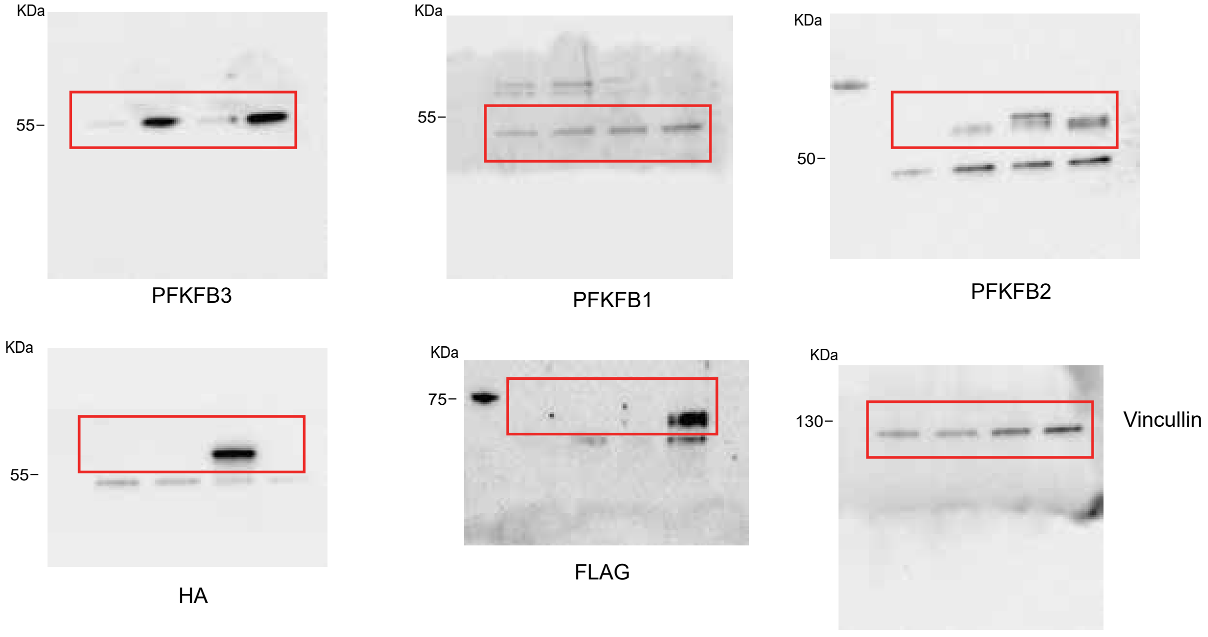

Loading from same gel

Extended Data Fig. 7f

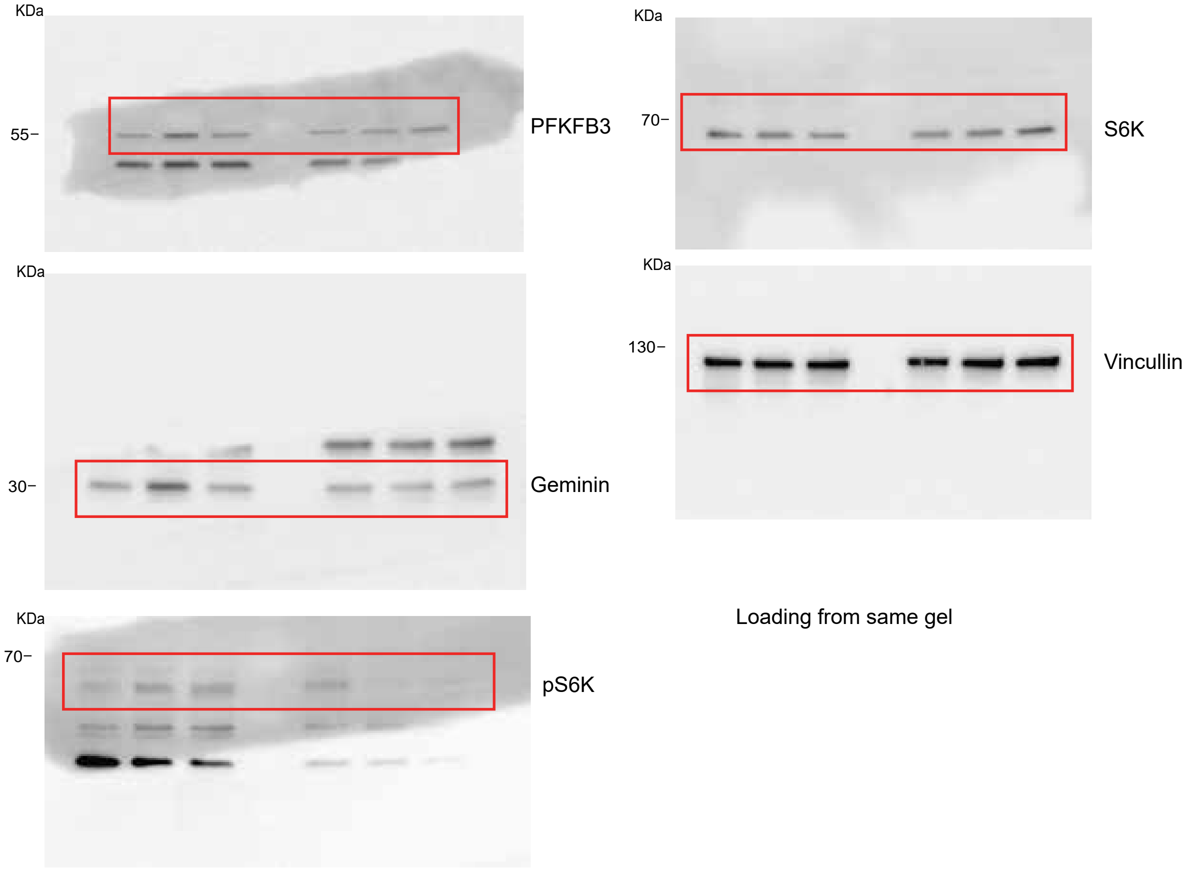

Loading from same gel

Extended Data Fig. 8a

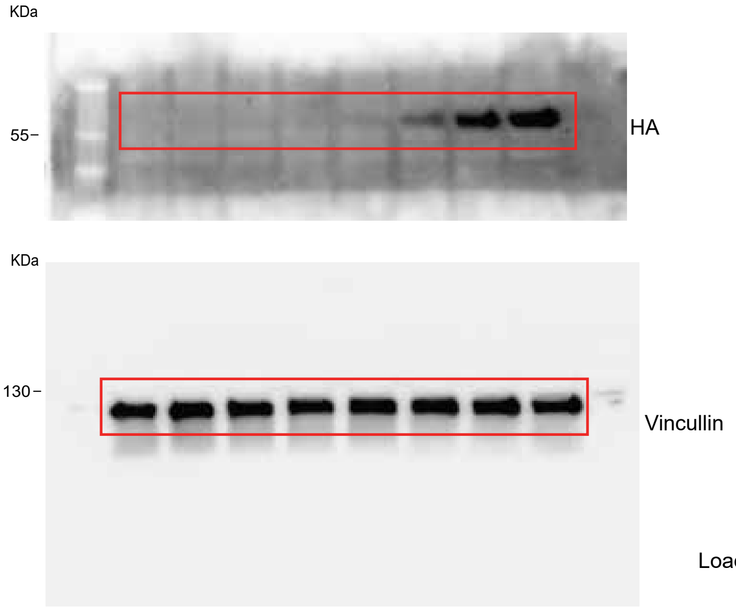

Loading from same gel

Extended Data Fig. 8b

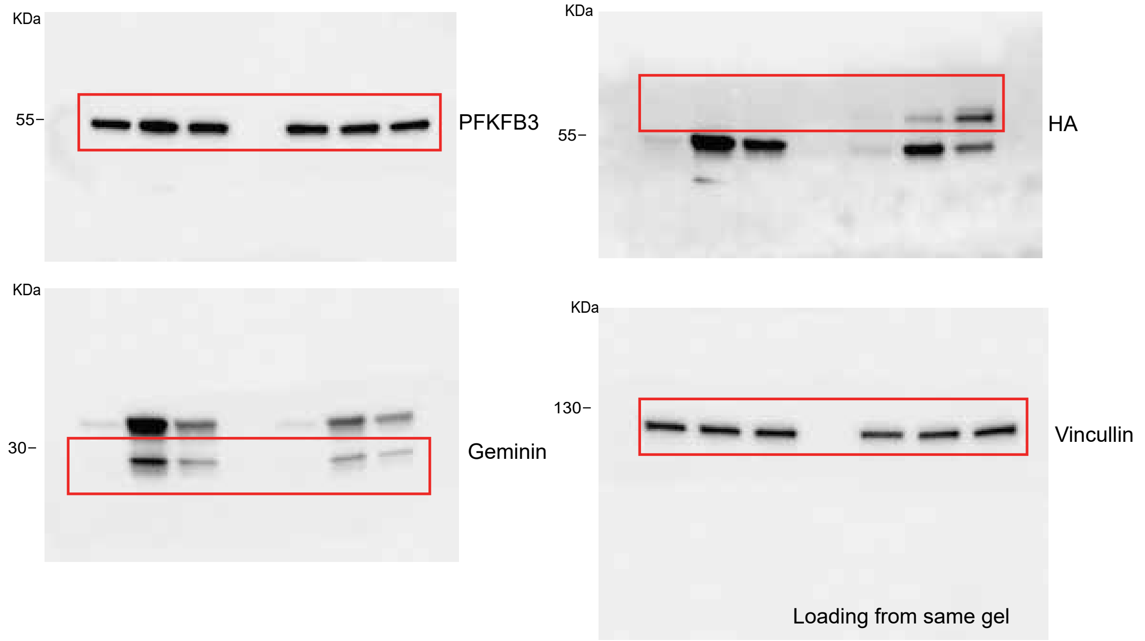

Extended Data Fig. 8d

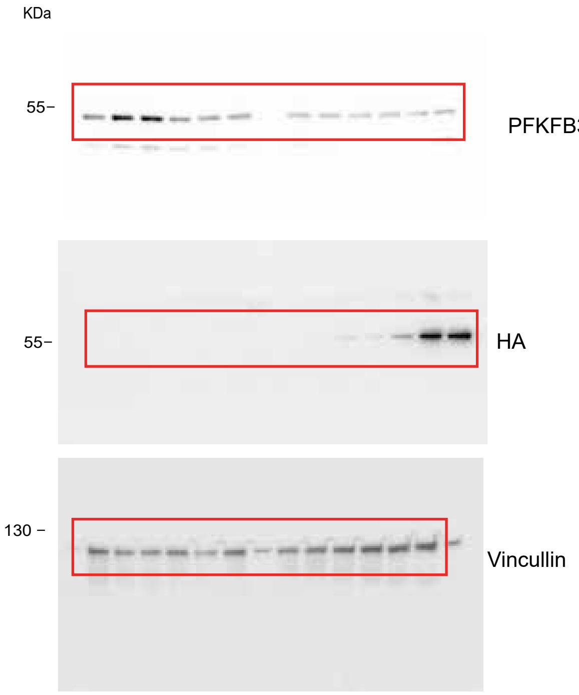

Loading from same gel

Extended Data Fig. 8f

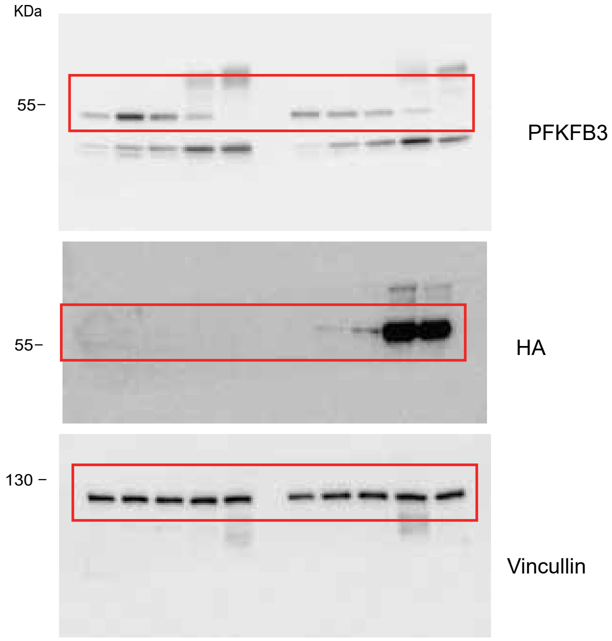

Loading from same gel

HA from a rerun gel

Extended Data Fig. 8h

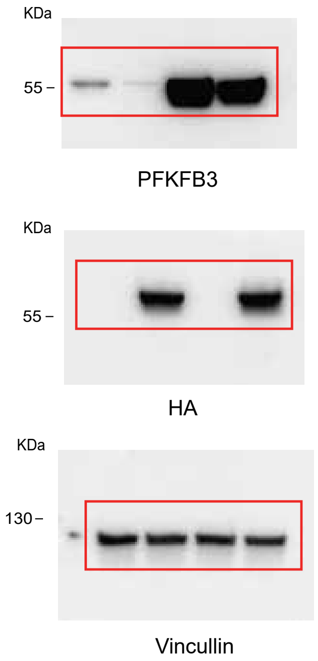

Loading from same gel

Extended Data Fig. 8k

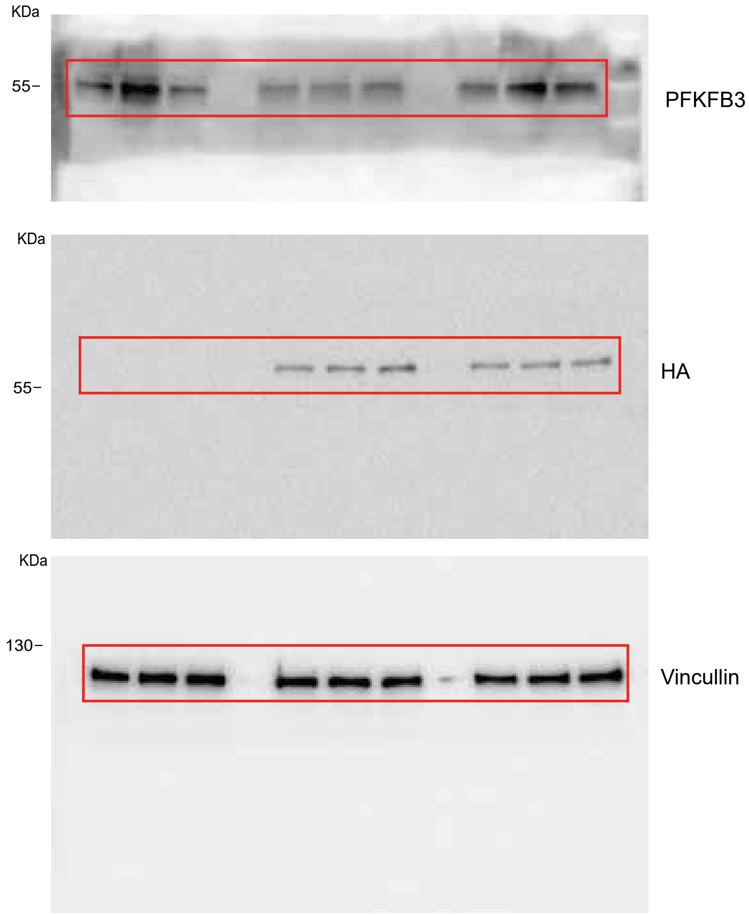

Loading from same gel

Extended Data Fig. 8i

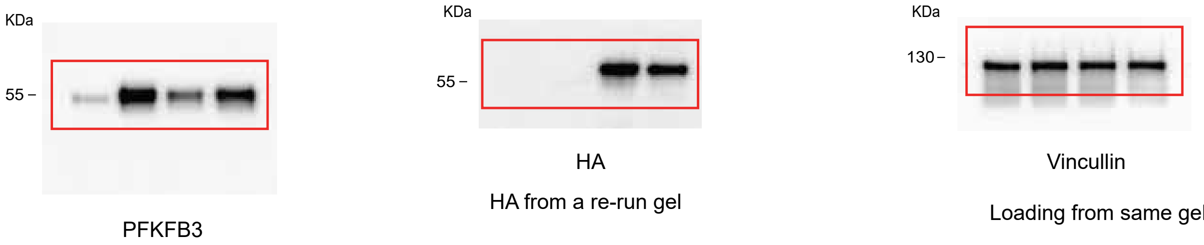

HA from a re-run gel

Loading from same gel

Extended Data Fig. 9g

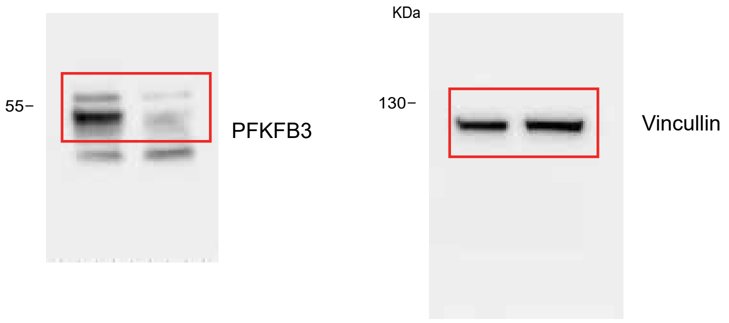

Extended Data Fig. 10a

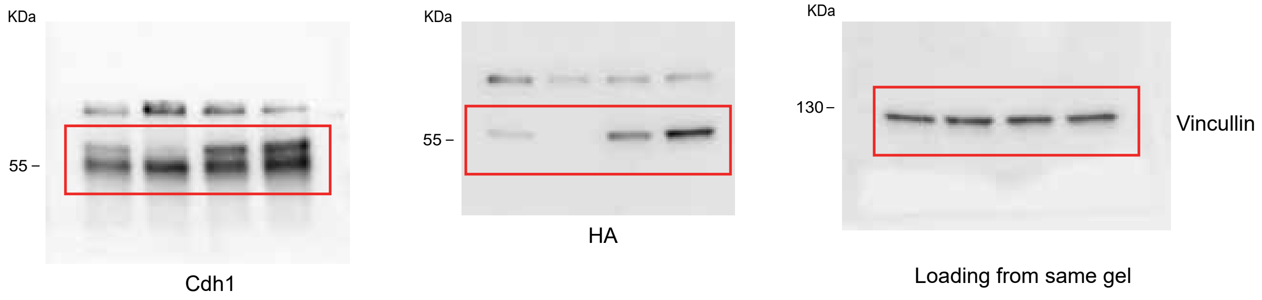

Extended Data Fig. 10f

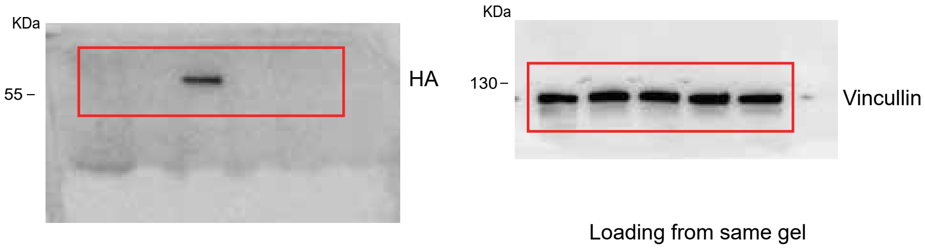

Supplement: Supplementary file 1 — All raw western blots. [file 41586_2025_9328_MOESM1_ESM.pdf]
