# Supplementary material for: Transient APC/C inactivation by mTOR boosts glycolysis during cell cycle entry
Source: Nature. 2025 Jul 30;646(8083):198–207. doi: 10.1038/s41586-025-09328-w (PMC12488482; doi:10.1038/s41586-025-09328-w)
Supplement: Supplementary file 2 — Reporting Summary [file 41586_2025_9328_MOESM2_ESM.pdf]

nature portfolio

Corresponding author(s): Steven D. Cappell
Last updated by author(s): Jun 12, 2025

Reporting Summary

Nature Portfolio wishes to improve the reproducibility of the work that we publish. This form provides structure for consistency and transparency in reporting. For further information on Nature Portfolio policies, see our Editorial Policies and the Editorial Policy Checklist.

Statistics

For all statistical analyses, confirm that the following items are present in the figure legend, table legend, main text, or Methods section.

n/a	Confirmed	
☐	☒	The exact sample size (*n*) for each experimental group/condition, given as a discrete number and unit of measurement
☐	☒	A statement on whether measurements were taken from distinct samples or whether the same sample was measured repeatedly
☐	☒	The statistical test(s) used AND whether they are one- or two-sided<br>*Only common tests should be described solely by name; describe more complex techniques in the Methods section.*
☒	☐	A description of all covariates tested
☒	☐	A description of any assumptions or corrections, such as tests of normality and adjustment for multiple comparisons
☐	☒	A full description of the statistical parameters including central tendency (e.g. means) or other basic estimates (e.g. regression coefficient) AND variation (e.g. standard deviation) or associated estimates of uncertainty (e.g. confidence intervals)
☐	☒	For null hypothesis testing, the test statistic (e.g. *F*, *t*, *r*) with confidence intervals, effect sizes, degrees of freedom and *P* value noted<br>*Give P values as exact values whenever suitable.*
☒	☐	For Bayesian analysis, information on the choice of priors and Markov chain Monte Carlo settings
☒	☐	For hierarchical and complex designs, identification of the appropriate level for tests and full reporting of outcomes
☒	☐	Estimates of effect sizes (e.g. Cohen's *d*, Pearson's *r*), indicating how they were calculated

Our web collection on statistics for biologists contains articles on many of the points above.

Software and code

Policy information about availability of computer code

Data collection	NIS Elements (v5.11.00)
Data analysis	MATLAB (vR2020b). Automated image analysis was performed using custom MATLAB scripts as described in Cappell, S.D. et al Cell 166, 167-180 (2016) (https://github.com/scappell/Cell_tracking). Graphpad Prism 9 (v9.2.0) was used for statistical analysis. RStudio (v1.3.1093) was used for mathematical modeling along with the deSolve package. Proteome Discoverer Software (v2.4) to analyze mass spec data. Xcalibur Quan Browser (v4.1) was used to analyze glucose tracing data.

For manuscripts utilizing custom algorithms or software that are central to the research but not yet described in published literature, software must be made available to editors and reviewers. We strongly encourage code deposition in a community repository (e.g. GitHub). See the Nature Portfolio guidelines for submitting code & software for further information.

Data

Policy information about availability of data

All manuscripts must include a data availability statement. This statement should provide the following information, where applicable:

- Accession codes, unique identifiers, or web links for publicly available datasets
- A description of any restrictions on data availability
- For clinical datasets or third party data, please ensure that the statement adheres to our policy

All data is available in the Source Data file. The datasets generated during and/or analyzed during the current study are also available from the corresponding author on reasonable request. All data supporting the findings of this study are available from the corresponding author on reasonable request. Data from the

glucose tracing experiment is available on MassIVE (https://massive.ucsd.edu/ProteoSAFe/static/massive.jsp?redirect=auth) with unique identifier MSV000098175.

Research involving human participants, their data, or biological material

Policy information about studies with human participants or human data. See also policy information about sex, gender (identity/presentation), and sexual orientation and race, ethnicity and racism.

Reporting on sex and gender	n/a
Reporting on race, ethnicity, or other socially relevant groupings	n/a
Population characteristics	n/a
Recruitment	n/a
Ethics oversight	n/a

Note that full information on the approval of the study protocol must also be provided in the manuscript.

Field-specific reporting

Please select the one below that is the best fit for your research. If you are not sure, read the appropriate sections before making your selection.

- [x] Life sciences
- [ ] Behavioural & social sciences
- [ ] Ecological, evolutionary & environmental sciences

For a reference copy of the document with all sections, see nature.com/documents/nr-reporting-summary-flat.pdf

Life sciences study design

All studies must disclose on these points even when the disclosure is negative.

Sample size	All sample sizes were chosen based on conventional standards in our fields, considering previously published results. See Chung et al., 2019 (https://doi.org/10.1016/j.molcel.2019.08.020), Cornwell et al., 2023 (https://doi.org/10.1038/s41586-023-06274-3), Franks et al., 2020 (https://doi.org/10.1371/journal.pbio.3000975), Kosaisawe et al., 20221 (https://doi.org/10.1016/j.cmet.2021.01.014).
Data exclusions	No data was excluded form the experiments.
Replication	All experiments in which p-values are present have been carried out with at least 3 replicates. All experiments were independently reproduced at least twice.
Randomization	Samples were allocated randomly for imaging and analysis. Representative single-cell traces where chosen at random from the population for visualization.
Blinding	Blinding was not relevant to this study. Image acquisition and analysis was conducted using automated scripts which are not subject to experimental bias. For western blots, blinding is not possible because samples need to be loaded in a particular order.

Reporting for specific materials, systems and methods

We require information from authors about some types of materials, experimental systems and methods used in many studies. Here, indicate whether each material, system or method listed is relevant to your study. If you are not sure if a list item applies to your research, read the appropriate section before selecting a response.

Materials & experimental systems

n/a	Involved in the study	
[ ]	[x]	Antibodies
[ ]	[x]	Eukaryotic cell lines
[x]	[ ]	Palaeontology and archaeology
[x]	[ ]	Animals and other organisms
[x]	[ ]	Clinical data
[x]	[ ]	Dual use research of concern
[x]	[ ]	Plants

Methods

n/a	Involved in the study	
[x]	[ ]	ChIP-seq
[x]	[ ]	Flow cytometry
[x]	[ ]	MRI-based neuroimaging

Antibodies

Antibodies used

Cdh1(FZR1) antibody (Santa Cruz; sc56312; 1:800)
PFKFB3 (Abcam, AB181861-1001, 1:4000)
Cdh1(FZR1) antibody (Abcam; ab217038; 1:1000)
mTOR antibody (Cell Signaling Technologies; #2972, 1:1000)
phospho mTOR (Cell Signaling Technologies; #291, 1:1000)
Ubiquitin (Santa Cruz; SC-8017; 1:800)
mouse anti-goat IgG-HRP (Santa Cruz; sc-2354; 1:10000)
Anti-rabbit IgG, HRP-linked Antibody (Cell Signaling Technologies; #7074; 1:10000)
Anti-mouse IgG, HRP-linked Antibody (Cell Signaling Technologies; #7076; 1:10000)
normal rabbit IgG (Cell Signaling Technologies; #2729, 2μg per IP)
normal mouse IgG (Santa Cruz; Sc2025, 2μg per IP).
mCherry (Abcam; ab167453; 1:1000)
Geminin (Cell Signaling Technologies; #5165; 1:1000)
Cyclin D1 (Thermo Scientific; MA5-14512; 1:750)
phospho Rb (Cell Signaling Technologies; #8516; 1:1000)
Rb (Cell Signaling Technologies; #9309; 1:2000)
Vinculin (Sigma; V9131; 1:10000)
Ki67(Abcam; ab8191;1:1000)
p21 (BD biosciences; 556430; 1:1000)
p27 (Cell Signaling Technologies; #3686; 1:1000)
His tag (Santa Cruz; Sc8036; 1:800)
anti-DDK/FLAG (Sigma; F3165; 1:1000)
phospho S6 Kinase (Cell Signaling Technologies; #9205; 1:1000)
APC2 (Cell Signaling Technologies; #12301; 1:1000)
APC6 (Cell Signaling Technologies; #9499; 1:1000)
APC11 (Cell Signaling Technologies; 14090; 1:1000)
S6 kinase (Cell Signaling Technologies; #9202; 1:1000)
phospho 4EBP1 (Cell Signaling Technologies; #2855; 1:1000)
Cyclin A2 (Santa Cruz; sc-271682; 1:500)
Emi1 (Santa Cruz; Sc-365212; 1:500)
Cyclin F (Santa Cruz; Sc-515207; 1:500)
βTrCP (Cell Signaling Technologies; #4394; 1:1000)
pSer/Thr/Tyr (Fisher; 61-8300; 1:1000)
pThr (Abcam; ab9337; 1:500)
pTyr (Abcam; ab10321; 1:1000)
HA (Santa Cruz; Sc-7392; 1:800)
HA (Cell Signaling Technologies; #3724; 1:1000)
GST (Santa Cruz; sc-138; 1:750)
PFKFB3 (MBS; 9604769; 1:750)
PFKFB2 (Cell Signaling Technologies; #13029; 1:1000)
PFKFB1 (Abcam; ab155564; 1:1000)
βActin (Abcam; ab6276; 1:2000)
GAPDH (Abcam; ab128915; 1:2000)
Raptor (Cell Signaling Technologies; #2280; 1:1000)
Rictor (Cell Signaling Technologies; #2114; 1:1000)
MSLT8 (Cell Signaling Technologies; #3274; 1:1000)

TSC1 (Cell Signaling Technologies ,#6935, 1:1000)
NPRL2 (Cell Signaling Technologies, #37344, 1:1000)
Histone H1 (Abcam, 11079, 1:1000)
EGFR (Santa Cruz, sc-373746, 1:800)
AKT (Cell Signaling technologies,#9272, 1:1000)
phospho AKT (Cell Signaling technologies ,#4060, 1:1000)
pan anti-pS/T antibody (Phospho Solutions, Cat. #PP2551; 1:2000)
CDH1 (Sigma, Cat. # CC43-100UG; 1:2000)
mouse anti-Rabbit secondary-HRP (SantaCruz Biotechnology, Cat. #sc-2357; 1:2500)
or recombinant anti-mouse (SantaCruz Biotechnology, Cat. #sc-516102; 1:2500)

Validation

All the antibodies used in this study are commercially available and extensively validated by the company, us, or others. Validation data is available in each of these company's website. In addition, we have confirmed the specificity of the following antibodies using siRNA-mediated knockdown and western blotting: Cyclin A2(ED Fig. 3b), Emi1 (ED Fig. 3c), Cyclin F (ED Fig. 3d), beta-TrCP (ED Fig. 3e), Cdh1 (alias FZR1, Fig. 4c, ED Fig. 5o), PFKFB3 (ED Fig. 9g), Raptor and Rictor (ED Fig. 3m). All other antibodies were not directly validated by us but were validated by the manufacturer for the same species and application as they were used in this study.
The validation for all the antibodies used are as follows- Cdh1 (FZR1) antibody (Abcam, Ab217038, 1:1000, IP: 2 μg) validated in this study and PMCID: PMC7505520, and (Santa Cruz, sc-56312, IB: 1:800) validated in PMID: 29160310 and PMID: 33523889, (Sigma,

Cat. # CC43-100UG; 1:2000) validated in PMID: 32345958 , mTOR antibody (CST, #2972, 1:1000) is validated by the manufacturer and PMID: 38886756, phospho mTOR (CST, #2971, 1:1000) is validated by the manufacturer and PMID: 38886756, mCherry (Abcam, ab167453, 1:1000) is validated by the manufacturer and PMCID: PMC10699776, Geminin (CST, #5165, 1:1000) in PMCID: PMC6390124, Cyclin D1 (Thermo Scientific, MA5 14512, 1:750) is validated by the manufacturer, phospho Rb (Ser807/811) (CST, #8516, 1:1000) by manufacturer and PMCID: PMC11208143, Rb (CST, #9309, 1:2000) by the manufacturer, Vinculin (Sigma, V9131, 1:10000) by manufacturer and , Ki67 (Abcam, ab8191, 1:1000) by PMID: 31707342, p21 (BD biosciences, 556430, 1:1000) by PMID: 38811535, p27 (CST, #3686, 1:1000) by the manufacturer, His tag (Santa Cruz, sc-8036, 1:800) by the manufacturer and PMID: 38848692, anti-DDK/FLAG (Sigma, F3165, 1:1000) by PMID:39705142, phospho S6 Kinase (CST, #9205, 1:1000) by manufacturer and PMID:39028622, PMID: 38886756, APC2 (CST, #12301, 1:1000) by manufacturer and PMID: 29987118, APC6 (CST, #9499, 1:1000) by manufacturer and PMID: 34626566, APC11 (CST, #14090, 1:1000) by manufacturer and PMID: 29987118, S6 kinase (CST, #9202, 1:1000) by manufacturer and PMID: 37083230, phospho 4EBP1 (CST, #2855, 1:1000) by PMID: 38124228, Cyclin A2 (Santa Cruz, sc-271682, 1:500) by PMID: 31380287, Emi1 (Santa Cruz, sc-365212, 1:500) by PMID: 28604711 and PMID: 28604711, Cyclin F (Santa Cruz, sc-515207, 1:500) by PMID: 36951214, βTrCP (CST, #4394, 1:1000) by PMID: 36973255, pSer/Thr/Tyr (Fisher, 61-8300, 1:1000) by manufacturer and PMID: 37874675, pThr (Abcam, ab9337, 1:500) in this study, pTyr (Abcam, ab10321, 1:1000) by manufacturer and PMID: 34697378, pan anti-pS/T antibody (Phospho Solutions, Cat. #PP2551; 1:2000) by PMID: 27841876, HA (Santa Cruz, sc-7392, 1:800, IP: 2 μg and CST, #3724, 1:1000) by manufacturer and PMID: 39627198, GST (Santa Cruz, sc-138, 1:750) by manufacturer and PMID: 40287465, PFKFB3 (MBS, 9604769, IB: 1:750, IP: 2 μg) by PMID: 36289220, PFKFB3 (Abcam, AB181861-1001, 1:4000) by manufacturer, PFKFB2 (CST, #13029, 1:1000) by manufacturer and PMID: 32718270, PFKFB1 (Abcam, ab155564, 1:1000) by PMID: 34679684, βActin (Abcam, ab6276, 1:2000) by manufacturer, GAPDH (Abcam, ab128915, 1:2000) by manufacturer and PMID: 38123554, Raptor (CST, #2280, 1:1000) by PMID: 35869262, Rictor (CST, #2114, 1:1000) by PMID: 35869262, MSLT8 (CST, #3274, 1:1000) by PMID: 20169205, Ubiquitin (Santa Cruz, sc-8017, 1:800) by PMID: 36746962, TSC1 (CST,#6935, 1:1000) by manufacturer and PMID: 36396656, NPRL2 (CST, #37344, 1:1000) by PMID: 36044864, Histone H1 (Abcam, 11079, 1:1000) by manufacturer, EGFR (Santa Cruz, sc-373746, 1:800) by PMID: 36795511, AKT (CST,#9272, 1:1000) by PMID: 40155685, phospho AKT (CST,#4060, 1:1000) by manufacturer and PMID: 40191596, mouse anti-goat IgG-HRP (Santa Cruz, sc-2354, 1:10000) by PMID: 40033150, Anti-rabbit IgG, HRP-linked Antibody (CST, #7074, 1:10000) by PMID: 39972131, mouse anti-Rabbit secondary-HRP (SantaCruz Biotechnology, Cat. #sc-2357; 1:2500) by PMID: 33397958 or recombinant anti-mouse (SantaCruz Biotechnology, Cat. #sc-516102; 1:2500) by PMID: 32341344, Anti-mouse IgG, HRP-linked Antibody (CST, #7076, 1:10000) by PMID: 40069201, normal rabbit IgG (CST, #2729, IP: 2 μg) by PMID: 28813410, normal mouse IgG (Santa Cruz, sc-2025, IP: 2 μg) by PMID: 26880551.

Eukaryotic cell lines

Policy information about cell lines and Sex and Gender in Research

Cell line source(s)	MCF10A (ATCC: CRL-10317)<br>RPE-1 (ATCC: CRL-4000)<br>HLF (ATCC: PCS-201-013)<br>HEK293T (gift from Dr. Tobias Meyer's Laboratory at Weil Cornell Medical School, ATCC: CRL3216)<br>Mouse MEF (gift from Dr. Joanna Vidigal's Laboratory at the National Cancer Institute)
Authentication	Cell lines purchased from ATCC were not further authenticated. HEK293T are not authenticated.
Mycoplasma contamination	Cells used in all experiments were routinely tested for mycoplasma contimination and only mycoplasma-negative cells were used in experiments
Commonly misidentified lines<br>(See ICLAC register)	No commonly misidentified cell lines were used in the study
